# High Level of Staufen1 Expression Confers Longer Recurrence Free Survival to Non-Small Cell Lung Cancer Patients by Promoting THBS1 mRNA Degradation

**DOI:** 10.3390/ijms23010215

**Published:** 2021-12-25

**Authors:** Florence Bonnet-Magnaval, Leïla Halidou Diallo, Valérie Brunchault, Nathalie Laugero, Florent Morfoisse, Florian David, Emilie Roussel, Manon Nougue, Audrey Zamora, Emmanuelle Marchaud, Florence Tatin, Anne-Catherine Prats, Barbara Garmy-Susini, Luc DesGroseillers, Eric Lacazette

**Affiliations:** 1 U1297-Institut des Maladies Métaboliques et Cardiovasculaires (I2MC), Institut National de la Santé et de la Recherche Médicale (INSERM), Université de Toulouse, F-31432 Toulouse, France; florence.bonnet@umontreal.ca (F.B.-M.); leila.diallo@inserm.fr (L.H.D.); valerie.brunchault@inserm.fr (V.B.); nathalie.laugero@inserm.fr (N.L.); florent.morfoisse@inserm.fr (F.M.); florian.david@inserm.fr (F.D.); emile.roussel@inserm.fr (E.R.); manon.nougue@inserm.fr (M.N.); audrey.zamora@inserm.fr (A.Z.); emmanuelle.marchaud@inserm.fr (E.M.); florence.tatin@inserm.fr (F.T.); barbara.garmy-susini@inserm.fr (B.G.-S.); 2 Département de Biochimie Et Médecine Moléculaire, Faculté de Médecine, Université de Montréal, 2900 Édouard Montpetit Montréal, Montreal, QC H3T 1J4, Canada; luc.desgroseillers@umontreal.ca

**Keywords:** SMD (Staufen-mediated mRNA decay), tumorigenesis, thrombospondin

## Abstract

Stau1 is a pluripotent RNA-binding protein that is responsible for the post-transcriptional regulation of a multitude of transcripts. Here, we observed that lung cancer patients with a high Stau1 expression have a longer recurrence free survival. Strikingly, Stau1 did not impair cell proliferation in vitro, but rather cell migration and cell adhesion. In vivo, Stau1 depletion favored tumor progression and metastases development. In addition, Stau1 depletion strongly impaired vessel maturation. Among a panel of candidate genes, we specifically identified the mRNA encoding the cell adhesion molecule Thrombospondin 1 (THBS1) as a new target for Staufen-mediated mRNA decay. Altogether, our results suggest that regulation of THBS1 expression by Stau1 may be a key process involved in lung cancer progression.

## 1. Introduction

Given that RNA-binding proteins (RBPs) play a critical role in ensuring that proteins involved in the same biological pathway are translated in a highly coordinated fashion, it is perhaps no surprise that recent research has linked RBPs to the modulations of cell survival and homeostasis. Indeed, RBPs are critical modulators of RNA metabolism and organize functionally related mRNAs into RNA regulons [1,2]. RNAs (mRNAs, miRNAs and/or lncRNAs) and RBPs associate in a spatio-temporal manner via a series of highly ordered interactions between specific mRNAs to form ribonucleoproteins (RNPs). RBPs regulate various processes such as transcription, splicing, polyadenylation, editing, translocation, translation and turnover of the targeted mRNAs, which will determine their fate in the cell [3]. Consequently, even a slight modulation in the expression/activity of a single RBP can deeply impact one or more pathway(s) by altering a large network of downstream regulated genes. Many studies report that mutations affecting the functions of RNA-binding proteins impair various biological networks and are frequently observed in several diseases including cancers [4,5,6]. Subsequently, many RBPs have been identified as critical players in the different steps of cancer development and progression. Among these RBPs, Stau1 appears as a key component of cancer development, as there is increasing evidence establishing a correlation between the deregulation of its expression and tumor progression.

Stau1 is a double-stranded RNA-binding protein acting as a multifunctional post-transcriptional regulator [7]. Through binding to specific RNA groups, Stau1 controls their translation [8,9,10,11,12], transport and localization [13,14], differential splicing [15,16], nuclear export [15,16] and decay [17]. Three isoforms are generated by alternative splicing, Stau1^55^, Stau1^55i^ and Stau1^63^ [18,19,20]. The differential roles of these isoforms remain unclear. Large-scale analyses have demonstrated that Stau1 binds a broad spectrum of mRNAs coding for functionally diverse proteins that can be clustered within specific pathways. Accordingly, Stau1 was shown to be involved in cell proliferation [21,22,23], embryogenic development [24,25,26,27,28], cell differentiation [29], apoptosis [30,31,32], stress granule regulation [33,34,35], synaptic plasticity [36,37], and RNA virus replication [38,39,40,41,42,43,44]. The pleiotropic characteristics of Stau1 are the result of the large diversity of Stau1-bound RNAs and the various subsequent post-transcriptional mechanisms that are triggered by Stau1. Interestingly, the location of the Stau1 binding site (SBS) on the targeted mRNA determines the fate of this RNA. When Stau1 is recruited to an SBS located in the 5′UTR or CDS region, it exclusively triggers mechanisms that regulate mRNA translation [8,9,10,45]. When bound in the 3′UTR, Stau1 can activate different regulatory mechanisms. Genome-wide studies coupled to ribosome profiling approaches reveal that the recruitment of Stau1 to an SBS localized in the 3′UTR of mRNAs can enhance their translation [10]. This observation was validated in vitro by the demonstration that E2F1 mRNA translation is increased as a consequence of Stau1 binding in its 3′UTR [22]. In contrast, Stau1 interaction with the 3′UTR of MyoD mRNA in quiescent muscle stem cells rather prevents its translation [46], which suggests that the fate of Stau1-bound mRNAs depends on the cellular context and/or the final composition of the Stau1-containing RNP. Furthermore, Stau1 recruitment at the 3′UTR of mRNAs can also trigger Staufen-mediated mRNA decay (SMD).

SMD is initiated by the association of Stau1 to inter- or intramolecular double-stranded RNA structures within the 3′UTR. The RNA helicase up-frameshift 1 (UPF1), which is best known for its role in the nonsense-mediated mRNA decay (NMD) pathway, is subsequently recruited to trigger degradation of the target mRNA [29,47,48,49,49,50,51,52,53]. Although the exact mechanism leading to the target decay is still unclear, new studies describe UPF2 as a cofactor that links Stau1 to UPF1 [54]. Molecules such as the double-stranded RNA-binding proteins ADAR1p110 [31], NF45-NF90 [23] and several lncRNAs [55] compete with Stau1 by binding and masking the SBS, thus preventing Stau1 binding and decay of a subset of mRNAs, as well as improving the stability of these mRNAs. Thereby, the location of the cis-acting SBS elements on mRNAs and the presence of trans-acting cofactors will elicit different, even opposite mechanisms of gene regulation, allowing the fine-tuning of the expression of concomitant genes. A defect in Stau1 expression may impair concomitant expression of multiple downstream mRNAs involved in specific pathways and have disastrous consequences for the maintenance of cell homeostasis.

An increasing number of studies show that misregulation of Stau1 expression affects the delicate balance between oncogene, tumor-suppressor, and pro- and anti-apoptotic gene expression and favors cancer development [7]. Studies have shown that Stau1 directly binds the E2F1 mRNA 3′UTR and enhances its translation. E2F1 is a key regulator of cell cycle transitions and is an oncogene [56,57,58,59,60,61,62]. In turn, E2F1 binds the Stau1 gene promoter and increases Stau1 expression. In addition, this loop is likely strengthened by enhancement of C-MYC translation upon binding of Stau1 to the C-MYC 5′UTR [45]. C-MYC transcriptionally upregulates E2F1 expression and facilitates cell growth [63,64]. Therefore, Stau1, E2F1 and C-MYC synergistically favor cell proliferation. Furthermore, E2F1 induces the transcription of the long non-coding RNA (lncRNA) *TINCR* in gastric carcinoma and promotes gastric cancer progression [65]. Interestingly, *TINCR* facilitates Stau1 recruitment to the 3′UTR of the cyclin-dependent kinase inhibitor CDKN2B mRNA, a potent growth inhibitor of cell cycle G1-G1/S checkpoints [66]. Multiple other misregulated lcnRNAs were also shown to modulate SMD activity in several cancers, including gastric cancer, glioma, glioma-derived cells, and colorectal cancer [32,52,55,65,67,68,69], as a consequence resulting in proliferation, migration and invasiveness of cancer cells. In addition, aberrant expression of several lncRNAs causes the misregulation of SMD activity, modifying angiogenesis, vasculogenic-mimicry and blood barrier permeability [69,70,71]. Therefore, a modification of Stau1 expression and/or activity has disastrous consequences since Stau1 is a key regulator of various mRNA regulons that are critical for cell cycle regulation, and their misregulation triggering cancer development and progression. 

In the present study, we show that Stau1 expression is upregulated in non-small cell lung cancer and that high Stau1 expression is associated with better survival rate of patients with lung cancer. Using shRNA as a means to downregulate Stau1 expression in the H460 lung cancer cell line, we showed that Stau1 depletion enhances cell migration and invasiveness in vitro and facilitates tumor development and metastasis in mice. Interestingly, expression of Thrombospondin1 (THBS1) mRNA, one of the previously identified targets in cancer progression, is significantly increased in Stau1-depleted cells. THBS1 belongs to the adhesion glycoprotein family that mediates a wide range of biological effects including cell adhesion and migration. In this study, we validate THBS1 mRNA as a new target of Stau1 and show that its expression is regulated by SMD.

## 2. Results

### 2.1. Patients with Lung Cancer Displaying High Stau1 Expression Have Longer Recurrence Free Survival Probability

**Figure 1:**
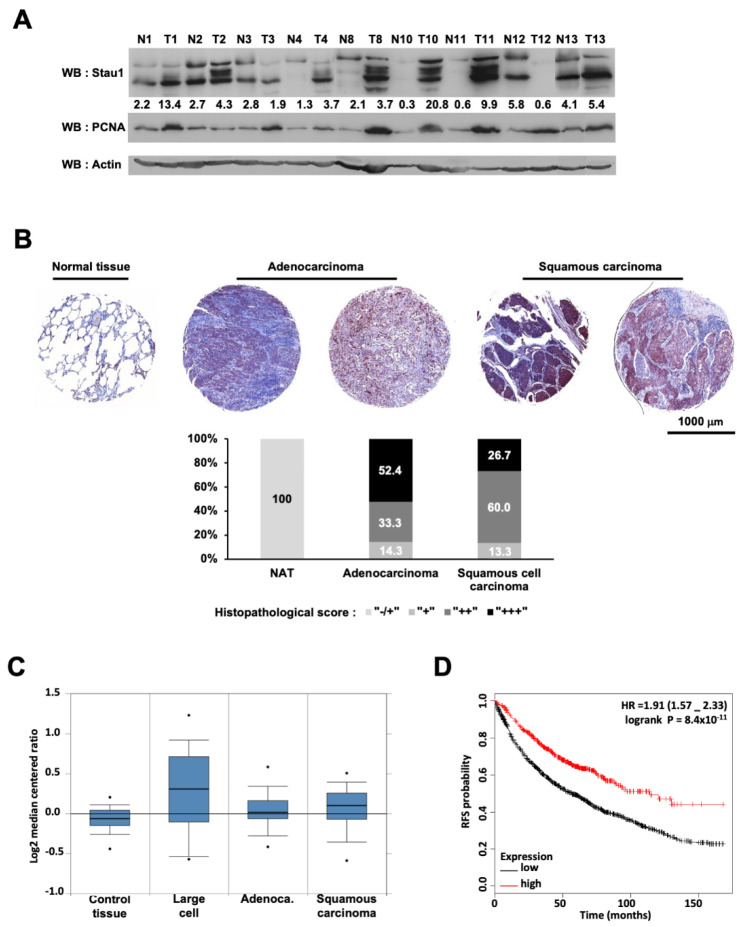
Stau1 is frequently upregulated in lung cancer and positively affects recurrence free survival. (**A**) Western blot analysis of protein lysates prepared from 9 matched samples of lung carcinoma tumors (T) and adjacent non-tumoral tissues (N). Equal amounts of protein from each pair were resolved on SDS-PAGE and immunoblotted with anti-Stau1, PCNA, and β-actin (loading control) antibodies. Several blots with the same exposure were assembled. Values represent Stau1 quantification normalized by actin quantification. Quantification was performed with Fiji Software. (**B**) Representative Stau1 immunostaining of normal lung tissue and 4 tumoral tissues, 2 adenocarcinomas and 2 squamous carcinomas from 2 tissue arrays containing triplicates of 5 adjacent/normal, 21 squamous cell carcinoma and 16 adenocarcinoma tissues from patients (LC722) and containing triplicates of 8 cases of squamous cell carcinoma, 12 adenocarcinoma, 2 lung large cell carcinoma, 1 each of atypical carcinoid and small cell carcinoma, plus 2 normal lung tissue (LC721). The graph represents the distribution in percent of histopathological score for the indicated tissues. (**C**) The level of Stau1 (mRNA) is shown using the Oncomine^TM^ gene expression data analysis tool and the data from the Hou study [72]. (**D**) Kaplan–Meier analysis for recurrence free survival in lung cancer patients according to the expression of Stau1. Auto select best cut-off was chosen for the analysis. The best specific Stau1 probe (JetSet probes) that recognized Affymetrix probe sets (213037_x_at) was chosen for the analysis. High levels of Stau1 expression were associated with recurrence free survival. The log-rank and the hazard ratio (HR) with 95% CI (Confidence Interval) is shown.

To establish a link between Stau1 expression and lung cancer, we first quantified Stau1 expression at the protein level in adjacent normal (N) and tumor tissues (T) of non-small cell lung patients (Figure 1A). Cell extracts from nine patients were analyzed by Western blotting. Seven of the nine extracts exhibited higher expression of Stau1 in tumors than in normal tissues whereas Stau1 expression was decreased in tumor samples from two patients (T3 and T8). Thus, Stau1 expression is misregulated in samples of patients with non-small cell lung cancer. Higher expression of Stau1 in lung adenocarcinoma and squamous carcinoma compared to normal tissue was also observed at the protein level by a qualitative immunohistochemistry analysis using anti-Stau1 antibody (Figure 1B), as 85.7% of adenocarcinoma and 86.7% of squamous carcinoma display a strong (++) to very strong (+++) staining for Stau1 (Supplementary Figure S1A). 

To investigate the clinical relevance of Stau1 expression level in non-small cell lung cancer patients, we performed a meta-analysis of published gene expression data using the Oncomine^TM^ database (Compendia Bioscience, Ann Arbor, MI, USA). To this aim, we compared Stau1 expression level of 156 samples of non-small cell lung cancer issued from the Hou study [72]. The sample batch was composed of 91 non-small cell lung carcinoma and 65 adjacent normal lung adjacent tissues (Figure 1C). Stau1 expression was on average 2.73-fold higher (*p* = 9.03 × 10^−4^) in large cell carcinoma compared to normal tissues and 2.22 (*p* = 1.80 × 10^−4^) and 2.16 (*p* = 2.33 × 10^−4^) fold higher in adenocarcinoma cells and squamous carcinoma cells (Figure 1C), respectively. Therefore, only modest variations of Stau1 mRNA expression were noticed between samples of adjacent normal tissues and lung tumor subtypes. However, we observed a larger dispersion of values in tumor samples than in healthy tissues. To make sense of these observations, we next examined the correlation between Stau1 expression and non-small cell lung cancer outcome using the online Kaplan–Meier plotter set of data (kmplot.com, accessed on 15 July 2021) [73]. This online tool allowed us to perform a meta-analysis of 1926 non-small cell lung cancer samples with the same JetSet probe (213037_x_at) as in the Hou study. As described above, we found two subpopulations of cancer cells showing either high or low Stau1 expression. Strikingly, we found that a high level of Stau1 was significantly associated with better survival rate in resection free survival outcomes (HR = 0.58; 95% CI = 0.5–0.68; *p* = 5.3 × 10^−12^) (Figure 1D). Indeed, patients with low Stau1 expression have a survival median of 57 months, while those with high Stau1 expression have a survival median of 114 months. The analysis of Stau1 expression in adenocarcinoma (HR = 0.38; 95% CI = 0.29–0.5; *p*= 3.5 × 10^−13^) (Supplementary Figure S1A), and on squamous cell carcinomas (HR = 0.76; 95% CI = 0.6–0.96; *p* = 0.023) (Supplementary Figure S1B) provided similar results, although the difference was less pronounced for squamous cell carcinomas. Taken together, these data indicate that a high level of Stau1 confers a better clinical outcome to non-small cell lung cancer patients particularly for the adenocarcinoma subgroup. Accordingly, we decided to further investigate Stau1 function at the cellular and molecular levels by using the non-small cell lung cancer cell line H460. 

### 2.2. Stau1 Depletion Does Not Impair Cell Proliferation

**Figure 2:**
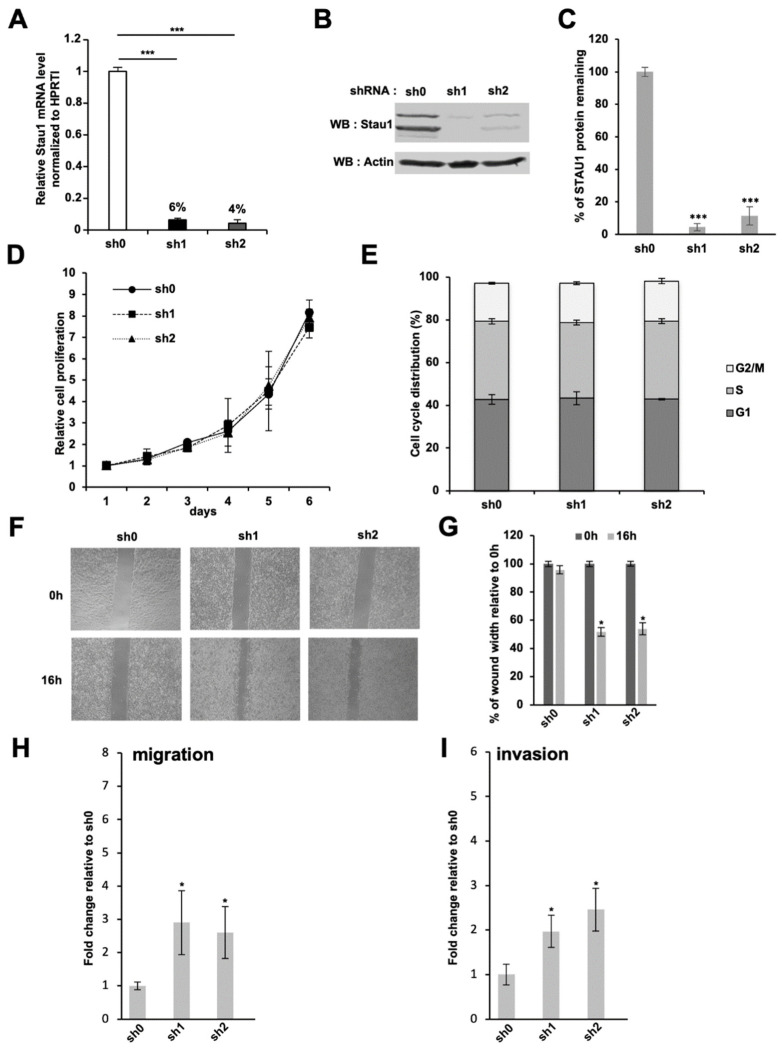
Effect of Stau1 knock-down in the H460 lung carcinoma cell line. (**A**) H460 cells were transduced by lentivectors expressing the indicated shRNA: sh0 has no target sequence in the human genome; sh1 and sh2 are two different shRNAs targeting the Stau1 coding sequence reducing Stau1 mRNA level by 94 and 96%, respectively. (**B**) Western blot analysis revealed that Stau1 protein levels are strongly impaired in sh1 and sh2 H460 cells compared to Stau1 sh0 control cell line. (**C**) Densitometry analysis on Stau1 protein remaining level relative to the sh0 control cell line was performed on 3 independent experiments and statistical analysis applied. (**D**) Cell proliferation analysis of the three indicated cell lines. (**E**) Cell cycle analysis at day 5. Stau1 depletion does not interfere with cell cycle progression in H460 cells. (**F**) Representative pictures of a wound healing assay performed on sh0, sh1 and sh2 cell lines after 16 h. (**G**) Quantification of (**F**). Stau1 depletion increases cell motility significantly. (**H**,**I**) Transwell assays were performed in Boyden chamber without (**H**) or with (**I**) Matrigel^TM^ coating to analyze the impacts of Stau1 depletion on the invasion and migration of lung cancer cells. Fold change relative to the sh0 control is shown. (Asterisks: * *p* < 0.05; and *** *p* < 0.001 in two-tailed Student’s *t*-test).

To get insight into Stau1 function during tumorigenesis, two stable knocked-down H460 cell lines were established using two distinct shRNAs (referred as sh1 and sh2) targeting Stau1 mRNA in exon 2, a sequence in the 5′UTR that is common to all Stau1 isoforms. A control H460 cell line, referred to as sh0, expressing a scrambled control shRNA, was also established. Using this approach, we obtained two Stau1-depleted H460 cell lines (Stau1-KD cells) that displayed 94% ± 0.14 and 96% ± 0.21 reduction in the amount of Stau1 mRNA for sh1 and sh2 cell lines, respectively, compared to the sh0 control cell line (Figure 2A). A similar reduction of Stau1 protein levels was observed by Western blotting (Figure 2B,C). Stau1 was almost undetectable in sh1 and sh2 cell lines (below 1% for sh1 and below 6% for sh2). 

Since Stau1 is an important regulator of the cell cycle, we then studied the impact of Stau1 depletion on cell proliferation using the growth curve assay. As shown in Figure 2D, no significant difference in cell proliferation was observed between the two Stau1-depleted cell lines and the control cell line, indicating that Stau1 expression is not essential for proliferation in the transformed H460 cell lines. A similar conclusion was obtained from the comparison of cell cycle phases using flow cytometry analysis (Figure 2E). Cell distributions in the G1, S and G2/M phases during exponential cell growth were similar in Stau1-depleted and Stau1-expressing cell lines. These results are consistent with previous studies that reported the absence of proliferation defect in Stau1-depleted cancer cells [21,74].

### 2.3. Stau1 Depletion Enhances Cell Migration of H460 Cells 

However, the features of tumor cells cannot be reduced to their proliferative capacity. Another important phenotype to assess the aggressiveness of a tumor resides in its capacity to perform the epithelial–mesenchymal transition (EMT) and the acquisition of migratory ability. To this end, we used the wound healing assay. Stau1-depleted and control H460 cells were seeded and when they reached 80 to 90% of confluence, a scratch was applied to create a wound. The gap that remained after 16 h was quantified. Quantification revealed that Stau1-KD cells filled approximately 50% of the wound area whereas the control cell line only filled 6% of the gap (Figure 2F,G), indicating that Stau1 depletion increases basal H460 cell migration. Since Stau1-depleted and control cell lines have identical proliferative capacities (Figure 2D,E), this increased wound healing ability of Stau1-KD cells is likely due to the acquisition of migratory properties. These results indicate that Stau1 expression prevents the migration of H460 cells. 

To confirm these results, Boyden chambers tests were also performed. Unlike the wound healing test, this test uses a gradient of chemotactic factors to determine the ability of the cells seeded in medium containing 0.5% serum in the upper chamber to migrate through a membrane to reach the bottom chamber containing 20% serum. Sixteen hours after plating, Stau1-depleted cells showed a 5.5 ± 1.4 (sh1) and 1.8 ± 0.2 (sh2) fold increase in the average number of migratory cells relative to the control cells (Figure 2H). These data indicate that the depletion of Stau1 protein leads to a strong increase in the migratory capacity of H460 cells by chemotactism. The Boyden chambers assay was reproduced with cells embedded in Matrigel^TM^ in the upper chamber. Our results showed that Stau1-KD cells had a significantly higher invasive ability compared to control cells: 3.3 ± 0.9 and 2.6 ± 0.7-fold increase for the sh1 and sh2 cell lines, respectively, compared to the control cell line (Figure 2I). In the light of these results, we concluded that Stau1 does not affect proliferation of H460 tumor cells but promotes anti-migratory and anti-invasive properties in H460 tumor cell lines.

### 2.4. Stau1 Knock-Down Inhibits Compact Tumor Spheroid Formation but Has No Effect on Anchorage-Independent Cell Growth

**Figure 3:**
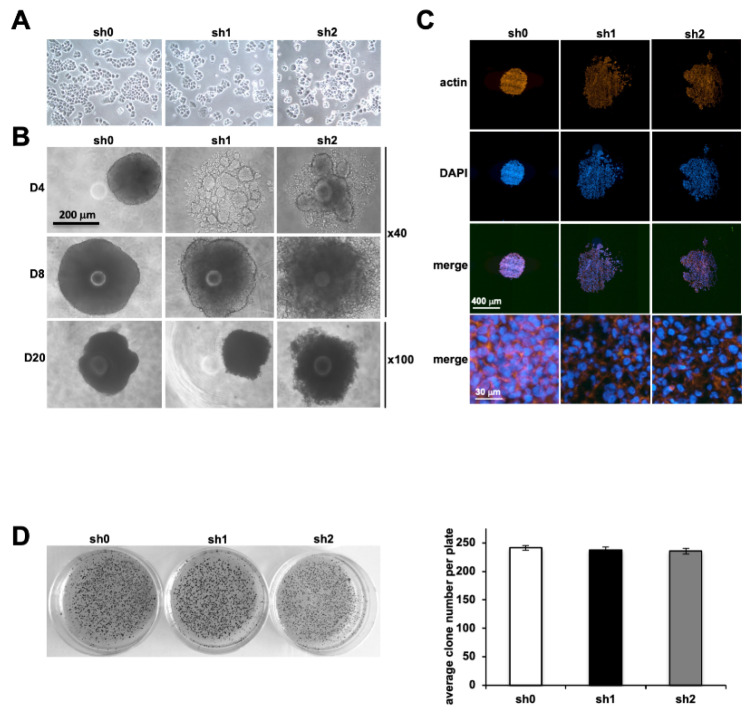
Effect of Stau1 depletion on tumor formation. (**A**) Representative pictures of sh0, sh1 and sh2 cell lines in 2D culture. Stau1 depletion has no visible impact. (**B**) Representative pictures of sh0, sh1 and sh2 cell lines on tumor spheroid formation at days 4, 8 and 20. Stau1 depletion prevents the formation of compact spheroid tumors in sh1 and sh2 cell lines. (**C**) Cross-sections were performed on spheroids. Actin cytoskeleton was evaluated and nuclei stained with DAPI. (**D**) Colony assay formation. Colonies were stained with MTT (left). Quantification did not reveal any significant difference between all three cell lines (right). Stau1 does not interfere with H460 cells’ capacities to form colonies.

In the 2D model of plated cells, no difference was observed in the morphological appearance and ability of Stau1-depleted cells to attach to the substrate compared to control cells (Figure 3A). However, in the spheroid formation assay in vitro that mimics 3D structure of micro-tumors, major morphological differences were detected between Stau1-depleted and control cells (Figure 3B). At day 4 after spheroid formation, the control cell line formed round-shaped compact spheroids, while the two Stau1-KD cell lines adopted an irregular and much looser structure. This phenotype was more pronounced with the sh2 cell line. These different phenotypes between the two Stau1-KD cells were also observed during spheroid development, although they appeared less pronounced at day 8 and day 20. These results tend to validate our previous observation regarding the lack of involvement of Stau1 on cell proliferation when grown in 3D, while clearly improving the intercellular interactions and adhesion since Stau1-KD cells were unable to form compact spheroids. Cross-sections on representative spheroids confirm that the structure is much looser in Stau1-KD cells lines than in the sh0 control cell line (Figure 3C). The actin cytoskeleton of Stau1-KD cells did not appear to be affected (Figure 3C, low row). DAPI staining confirmed that the cells were not apoptotic regardless of the Stau1 status of the cells. 

Subsequently, we evaluated the ability of isolated Stau1-KD cells to form colonies in vitro without anchoring by performing a soft agar assay colony formation. A representative result is shown in Figure 3D. Quantification of the number of colonies did not present any significant difference between the sh0 control cell line and the Stau1-KD cell lines (Figure 3D). Therefore, these results indicate that Stau1 did not interfere with the ability of H460 cells to grow independently of any anchorage. Thus, even if the Stau1-KD cells clearly exhibit cellular–cellular adhesion defects this has no functional consequence on the transformed phenotype of the H460 cells. Yet, according to our results, we hypothesize that cells expressing a lower level of Stau1 may present increased metastatic properties compared to cells expressing a higher level.

### 2.5. Stau1 Depletion Favors Tumor Progression and Metastasis Development

**Figure 4:**
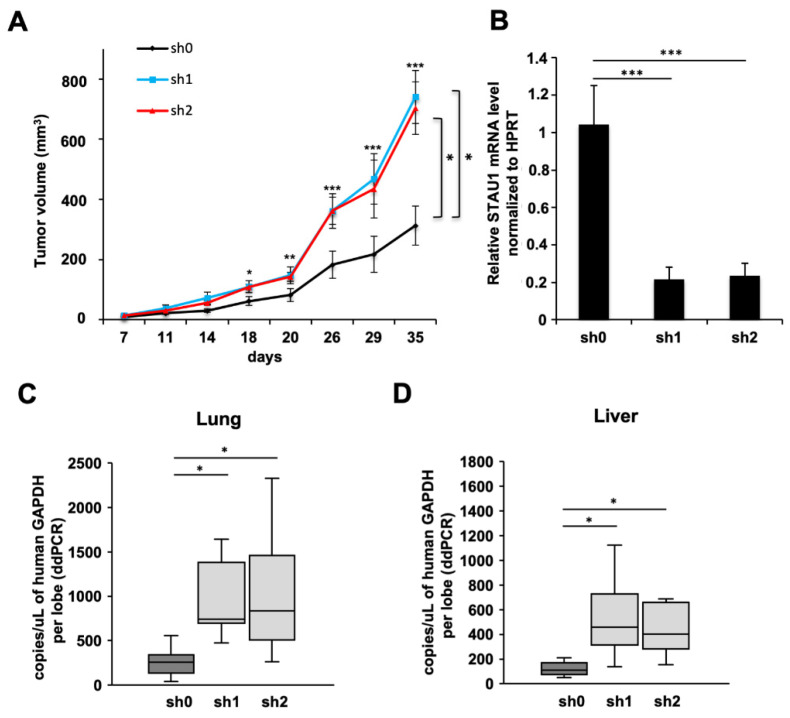
Stau1 depletion favors tumor progression and metastasis properties. (**A**) Tumor volumes measured at the indicated time points after subcutaneous injection of Stau1-deficient or control H460 cells into nude mice (statistic test: ANOVA + Turkey correction. sh0 vs. sh1 *p* = 0.0106; sh0 vs. sh2 *p* = 0.0244; sh1 vs. sh2 *p* = 0.965. * *p* < 0.05). Error bars show SEM. (**B**) Tumors were collected after 35 days and Stau1 mRNA level was controlled by RT-PCR after RNA extraction. (**C**,**D**) Tumor metastasis was evaluated by ddPCR by measuring human GAPDH mRNA levels in lung (**C**) and in liver (**D**). (Asterisks: * *p* < 0.05; ** *p* < 0.01 and *** *p* < 0.001 in two-tailed Student’s *t*-test).

Therefore, we evaluated the consequences of a low expression level of Stau1 in an in vivo context. To this purpose, the Stau1-KD and sh0 control cell lines were injected into nude mice and tumor volume was monitored for 35 days (Figure 4A). Strikingly, the results showed significantly larger tumor volume at day 35 for tumors initiated with Stau1-KD cell lines than for the sh0 control cell line (741 ± 88 mm^3^ and 703 ± 87 for sh1 and sh2, respectively, compared to 312 ± 65 mm^3^ for sh0) (Figure 4A). This suggests that the in vivo context plays a major role in tumor growth and that the tumor microenvironment of stromal origin influences the proliferation of Stau1-KD cells in vivo. As control, we showed that Stau1 expression is still knocked-down in tumors at day 35 (Figure 4B). However, because Stau1 regulates various genes involved in cell–cell and cell–matrix interactions, as well as cell proliferation, it is difficult to assess which mechanism is responsible for the observed phenotypes.

In order to determine if the differences between sh0 and the two Stau1-KD groups are dependent on proliferation rate and/or tumoral implantation, we performed a linear regression using the natural logarithm (ln) of tumor volume values (Supplementary Figure S2). The comparison of the slopes between the three groups suggests that the difference between tumor growth induced by sh0 cell line compared to Stau1-KD is due to a better tumor implantation in the first steps of tumor development rather than a difference in proliferation rate according to previous results (Figure 2D).

We then evaluated tumor metastasis in these three groups of mice. We took advantage of the method describe by Dahn et al. [75] by measuring GAPDH mRNA copy numbers originating from the tumors with validated human specific primers by RT-ddPCR in different mouse tissues to evaluate metastasis. The result presented in Figure 4C shows that the number of human GAPDH mRNA copies per microliter per lung lobe was significantly higher in the sh1 and sh2 groups compared to the sh0, with 1143 and 1052 copies/μL for sh1 and sh2, respectively, compared to 259 copies/μL on average. The same observation was made in liver even if the number of human GAPDH mRNA copies was lower than in lung (Figure 4D), i.e., 545 and 572 copies/μL for sh1 and sh2, respectively, compared to 173 copies/μL for sh0 in average. Altogether, our in vivo results demonstrated that the number of H460 cells which acquired the ability to disseminate into mice lungs and liver was significantly higher when Stau1 was depleted. 

### 2.6. Stau1 Depletion Impairs Tumor Vessels Maturation In Vivo

**Figure 5:**
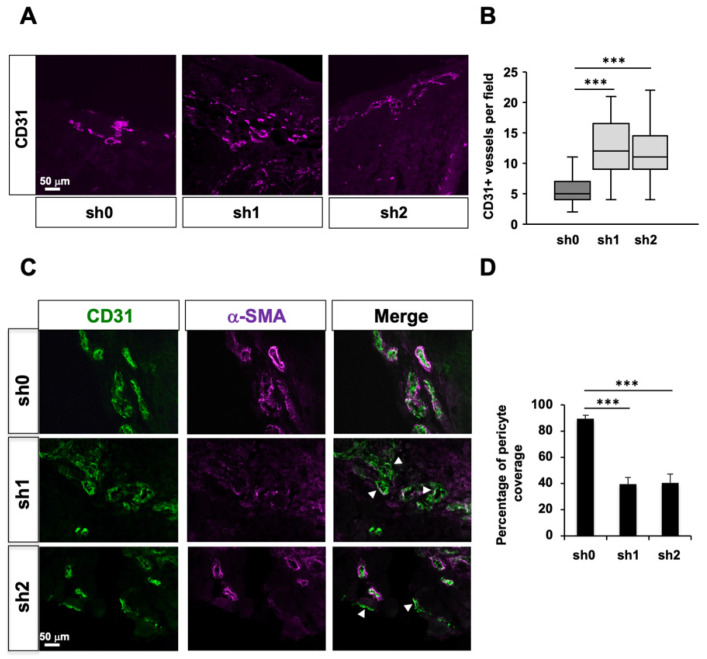
Stau1 depletion affects tumor angiogenesis. (**A**) CD31 staining on cross-sections of H460 tumors expressing sh0, sh1 and sh2 shRNAs. (**B**) Stau1 knock-down increased the number of blood vessels as shown by the quantification. (**C**) Maturity of blood vessels was evaluated by α-SMA and CD31 immunostaining (white arrows show blood vessels) and (**D**) quantification of pericyte coverage revealed a lack of pericyte coverage of tumor blood vessels irrigating Stau1-KD tumors (sh1 and sh2). The analysis was carried out on 5 mice for each of the 3 groups for which 5 fields were counted for every 6 slices observed. *** *p* < 0.001.

We then measured the number of blood vessels in tumors by counting CD31^+^ endothelial cell staining per fields (Figure 5A). Interestingly, the number of blood vessels was significantly increased in Stau1-KD sh1 and sh2 tumors compared to control sh0 tumors (Figure 5B), indicating an angiogenic effect as a consequence of Stau1 depletion. However, the number of blood vessels does not necessarily correlate with a performant tumor perfusion network. Indeed, the immature blood vessels network poorly irrigates tumor cells because they lack pericyte coverage, are more permeable and create a micro-environmental stress that favors EMT [76]. Therefore, we evaluated the maturity of blood vessels in Stau1-KD and normal tumors using IF staining with α-SMA (Smooth Muscle Actin) and CD31 staining (Figure 5C). Our results showed a strong decrease in α-SMA staining in both Stau1-KD sh1 and sh2 tumors (Figure 5D), revealing a significant lack of pericyte coverage on the blood vessels (white arrows). This result is consistent with the pro-metastatic phenotype of Stau1-depleted cells described above. 

### 2.7. Stau1 Depletion Impairs Expression of Genes Involved in Cell Adhesion

**Figure 6:**
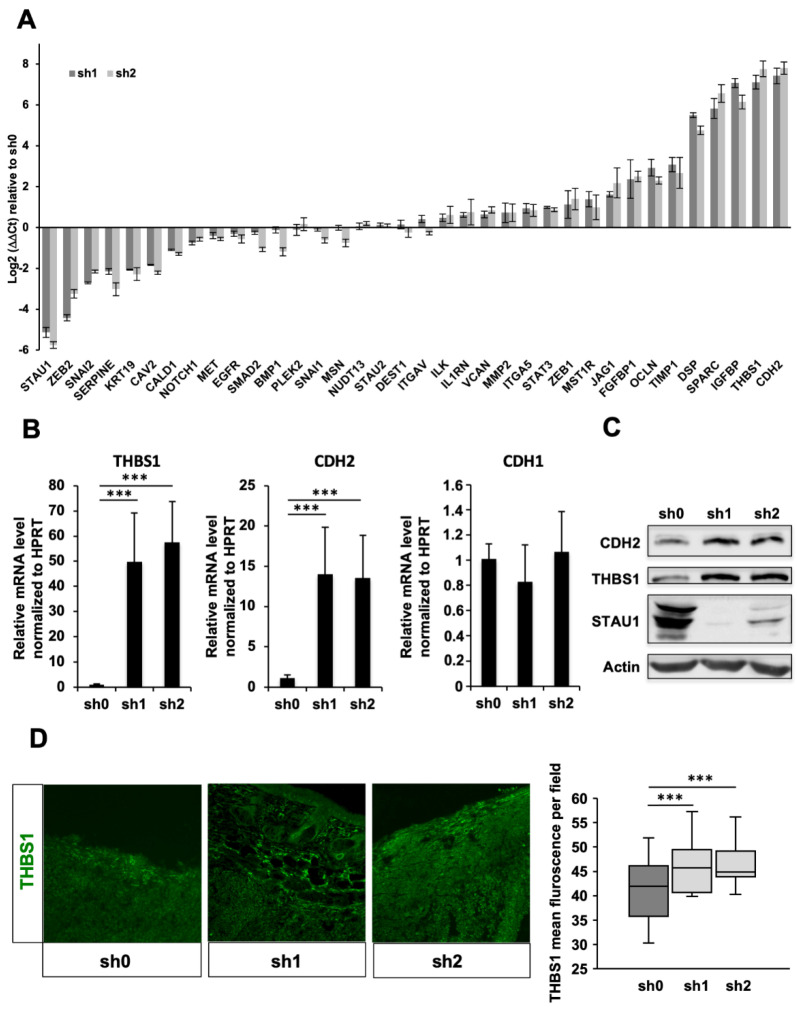
THBS1 and CDH2 expression is strongly increased by Stau1 depletion. (**A**) mRNA level of 35 genes involved in intercellular adhesion, cell adhesion to the extracellular matrix and EMT in sh1 and sh2 H469 cell lines compared to sh0 control cell line. Normalization was performed using HPRT gene. (**B**) THBS1 (left) and CDH2 (middle) mRNA levels are affected by Stau1 knock-down in xenografted tumors whereas CDH1 transcript levels remain unaffected (right). (**C**) The increase of THBS1 and CDH2 mRNA levels corresponds with an increase in THBS1 and CDH2 protein levels in the Stau1 knock-down cells sh1 and sh2 compared to the sh0 control cell line. (**D**) THBS1 protein levels are increased in Stau1 knock-down tumors sh1 and sh2 compared to sh0 control tumors. *** *p* < 0.001.

To understand these phenotypes at the molecular level, we investigated whether Stau1 depletion affects the expression of genes involved in intercellular adhesion, cell adhesion to the extracellular matrix and EMT. Therefore, we determined the expression of a panel of 35 genes in the above-mentioned processes that may be direct or indirect Stau1 target genes (Figure 6A) both in Stau1-KD and control H460 cell lines. Expression of at least 12 of these genes was misregulated (>4-fold, *p*-value < 0.05) in Stau1-KD compared to control cells. Among them, the amount of transcripts coding for THBS1 and CDH2 was increased more than 100-fold in the Stau1-KD cells compared to the control cell line. To confirm these results, we quantified the amount of THBS1 and CDH2 mRNAs in xenografted tumors. We also quantified the amount of CDH1 mRNA to determine if the ratio E-cadherin (CDH1)/N-cadherin (CDH2) is modified upon STAU1 depletion. The cadherin switch is one of the aspects of EMT and is known to profoundly affect tumor invasiveness and metastasis. Figure 6B showed an increase of 50-folds and 14-folds for THBS1 and CDH2 mRNAs, respectively, in Stau1-KD xenografted tumors compared to control tumors. In contrast, CDH1 expression remained unaffected by Stau1-KD tumors (Figure 6B), highlighting an increase in the CDH2/CDH1 ratio. It is likely that the overexpression of THBS1 and CDH2 in Stau1-KD cell lines and tumors induces defects of intercellular adhesion responsible for the phenotypes observed above (Figure 3A,B).

We then investigated whether the increase in the amounts of transcripts correlates with an accumulation of the proteins. Western blot analysis revealed a significant increase in the amount of THBS1 and CDH2 proteins in the Stau1-KD cell lines compared to the sh0 control cell line (Figure 6C), consistent with an increase at the transcript level. Similarly, using immunofluorescence analysis on tumor slices, we showed that the amount of THBS1 protein was significantly enhanced in Stau1-KD tumors compared to control tumors (Figure 6D).

**Figure 7:**
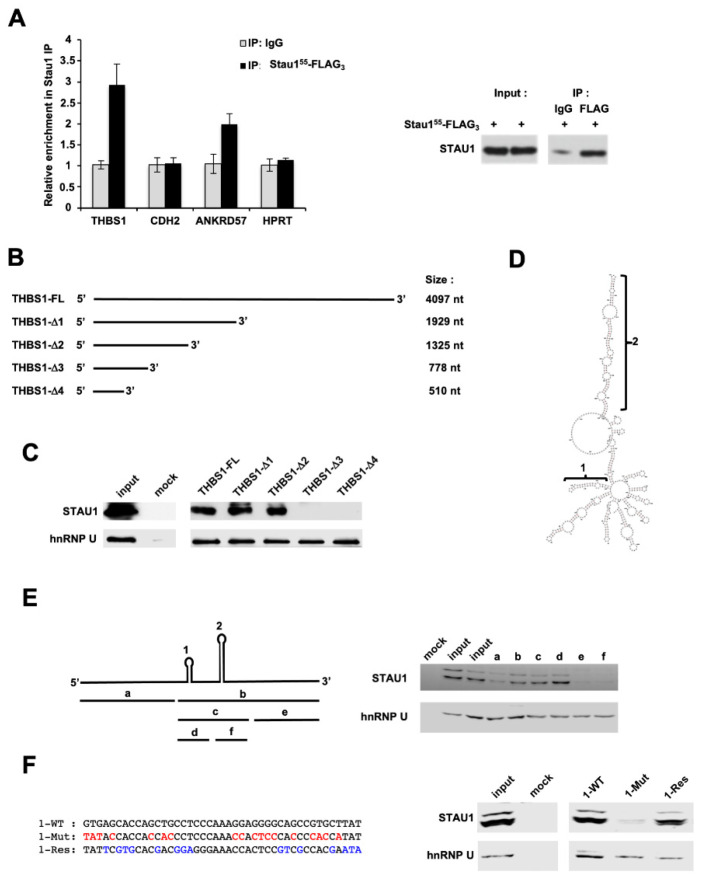
Stau1 binds to THBS1 mRNA. (**A**) RNA immunoprecipitation of THBS1 mRNA by Stau1, but not CDH2 mRNA (left). ANKRD57 and HPRT were used as a positive and a negative control, respectively. Western blot analysis was performed to control immunoprecipitation (right). (**B**) Deletion constructs of THBS1 3′UTR mRNA used to map the Staufen Binding Site (SBS). (**C**) Stau1 pull-down by the indicated portions of THBS1 3′UTR mRNA. (**D**) Prediction of the 547 nt sequence containing the SBS. (**E**) Precise mapping of Stau1 SBS in the “b” portion on the 547 nt fragment by RNA pull-down. (**F**) Mutagenesis confirmed that the “b” portion corresponds to the SBS as Stau1 did not bind to the 1-Mut construct, but 1-Res restored Stau1 binding.

THBS1 and CDH2 mRNAs contain long 3′UTRs able to form multiple potential intramolecular double-stranded Stau1-binding structures (Supplementary Figure S3A,B). We thus used RIP-qPCR experiment to determine if THBS1 and CDH2 mRNAs bind Stau1 in vitro. ANKRD57 (Ankyrin repeat domain 57) [50] and HPRT mRNAs were used as positive and negative controls, respectively. Our results showed an enrichment of nearly three-fold in the amount of THBS1 transcript in the Stau1-dependent immunoprecipitated group compared to the control IP (IgG) (Figure 7A), indicating that Stau1 directly binds THBS1 mRNA. Unexpectedly, CDH2 mRNA was not enriched in the IP, although it was previously identified in Stau1-CLIP in HEK 293 T cells [10].

### 2.8. The THBS1 mRNA 3′UTR Contains an Intramolecular Hairpin Structure That Recruits Stau1

In order to identify the binding site of Stau1 on the 3’ UTR of THBS1, pull-down RNA experiments were carried out using five biotinylated RNAs containing progressive deletions in the THBS1 3′UTR (Figure 7B). RNAs were transcribed in vitro, incubated in the presence of cytoplasmic extracts of H460 cells and the presence of Stau1 was visualized by Western blotting using anti-Stau1 antibody. Anti-hnRNP U was used as control. Our results indicated that the Stau1 binding site is located between nucleotides 778 and 1325 (Figure 7C). To more precisely map the SBS on THBS1 mRNA, the structure of the 547 nucleotides sequence was predicted using the RNA-fold software (Figure 7D). RNA-fold predicted the presence of two stable double-stranded stems. Comparison with the predicted structures of the truncated and full-length 3′UTR (Supplementary Figure S3B) revealed that only the small stem loop structure is common to both sequences and that the long stem loop does not form in the context of the full 3′UTR. To precisely map the location of the SBS, six additional transcripts were synthesized in vitro, corresponding to the regions depicted in Figure 7E, left, and named a to f. The pull-down assay showed that in vitro transcripts “b”, “c” and “d” were able to bind Stau1, suggesting that the small stem loop constitutes the SBS (Figure 7E, right). To confirm this conclusion, mutations were introduced in the “d” transcript to disrupt the double stranded stem (Figure 7F, left). As expected, we observed a loss of Stau1 binding when the stem was destroyed (1-Mut) compared to the wild-type sequence (1-WT) (Figure 7E, right) (Supplementary Figure S4, Mut vs. WT). We then generated additional mutations that restored the stem loop structure but not the original sequence to confirm that the stem loop structure formed by the “d” sequence is indeed necessary for Stau1 binding (1-Res) (Figure 7F, left) (Supplementary Figure S3, Res vs. WT). This transcript partially restored Stau1 recruitment (Figure 7F, right), thus proving that the small stem loop corresponds to the SBS on the THBS1 3′UTR mRNA. 

### 2.9. THBS1 Is a Target of the Stau1 mRNA-Mediated Decay 

**Figure 8:**
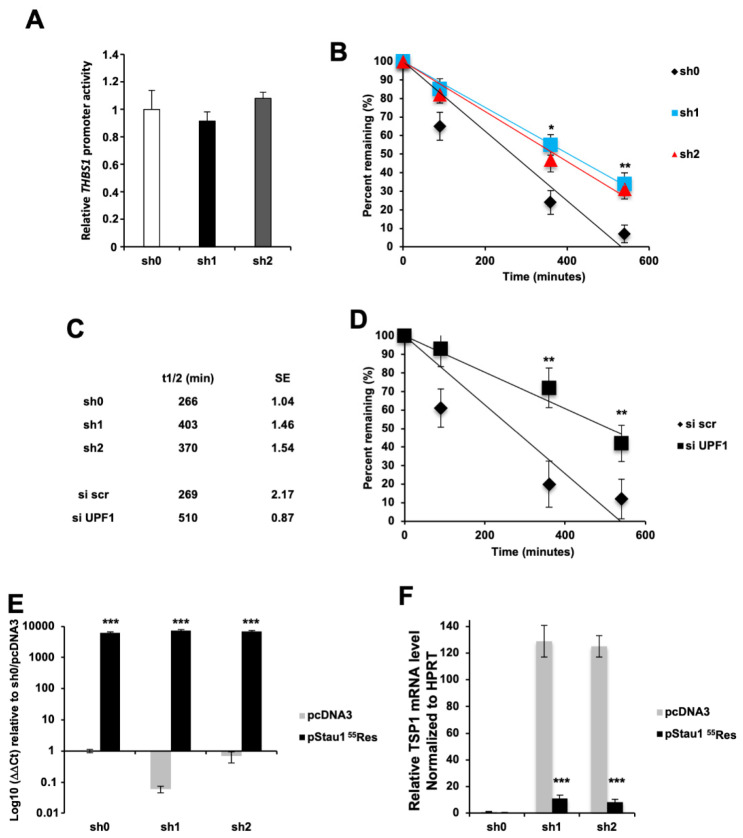
THBS1 is degraded by Staufen1 mRNA decay. (**A**) THBS1 promoter activity is not affected by Stau1-KD in H460 cells. (**B**,**C**) THBS1 transcript half-life is increased by Stau1 knock-down. (**D**) THBS1 transcript half-life is increased by UPF1 knock-down. (**E**) Stau1 expression is rescued in sh1 and sh2 H460 cell lines by a plasmid encoding a Stau1 mRNA resistant to sh1 and sh2 mRNA degradation (pStau1^55^Res) when compared to empty vector (pcDNA3). (**F**) THBS1 mRNA levels are strongly reduced when Stau1 is re-expressed in sh1 and sh2 cells. (Asterisks: * *p* < 0.05; ** *p* < 0.01 and *** *p* < 0.001 in two-tailed Student’s *t*-test).

To decipher the mechanism involved in THBS1 mRNA upregulation upon Stau1 depletion, we first determined if transcription of the THBS1 gene is enhanced in Stau1-depleted cells. For this, we used a plasmid containing the Firefly luciferase reporter gene under the control of the THBS1 promoter region (generous gift of F. Cabon). Co-expression of a second plasmid expressing the Renilla luciferase reporter expressed under a constitutive SV40 promoter allowed us to measure the ratio of both luciferases’ activities. No activation of the THBS1 promoter was observed in the Stau1-KD cell lines compared to the sh0 control cell line (Figure 8A), suggesting that the increase in the amount of THBS1 transcript in Stau1-KD cells lines was not due to an activation of THBS1 transcription.

Alternatively, upon binding in the 3′UTR of THBS1 mRNA, Stau1 could induce its degradation through SMD. To test this possibility, we blocked transcription in H460 cells with actinomycin D, an inhibitor of RNA polymerase II, and monitored the decay of THBS1 transcript over time in Stau1-KD and control H460 cell lines (Figure 8B). The half-life of the THBS1 transcript increased in the two Stau1-KD cell lines (403 and 370 min) compared to that in the control cell line (266 min) (Figure 8C). To prove the importance of SMD in THBS1 mRNA stability, we repeated this experiment in the presence of siRNA against UPF1, an essential cofactor of SMD. Results presented in Figure 8D show that the half-life of the THBS1 transcript was sensitive to Upf1 knock-down (siUPF1), as its half-life increased from 269 min to 510 min compared to the control (siSCR) condition (Figure 8C), indicating that THBS1 mRNA is a target of the SMD. Finally, to demonstrate that the effect observed on the THBS1 transcript stability is a direct consequence of Stau1 binding onto the THBS1 transcript, we overexpressed Stau1^55^ by transfecting a plasmid vector (pStau1^55^Res) carrying silent mutations that prevents sh1 and sh2 targeting, thus restoring expression of Stau1^55^ in the sh1 and sh2 Stau1-KD cell lines. The plasmid pStau1^55^Res, which was successfully expressed in all cell lines and rescued Stau1 expression (Figure 8E), strongly decreased the amount of THBS1 mRNA in the Stau1-KD cell by 91.27% and 93.47% (Figure 8F), confirming the involvement of Stau1 in the post-transcriptional regulation of THBS1 transcript. Altogether, these results validate THBS1 mRNA as a new target of Staufen1 mRNA-mediated decay.

Functionally, it is particularly interesting to note that a plasmid overexpressing THBS1 in NCI-H460 cells results in a similar phenotype in spheroid formation to that observed in cells expressing a shRNA directed against Stau1 (Supplementary Figure S5C). This result suggests that the effect observed by Stau1 depletion in sh1 and sh2 cell lines is mainly mediated by THBS1. Furthermore, a similar result was confirmed by the wound healing assay experiments. In this case, overexpression of THBS1 in NCI-H460 sh0 cells (sh0 + THBS1ov) resulted in an increased cell migration compared to sh1 and sh2 NCI-H460 cell lines, at 23.25% and 25.34, respectively (Supplementary Figure S5D,E). As expected, a pool of siRNAs directed against THBS1 (sh1 or sh2 + siTHBS1) partially reversed the phenotype observed with sh1 and sh2 (Supplementary Figure S5D,E). The relatively moderate effect observed is consistent with the partial decrease of THBS1 expression (43% of THBS1 mRNA expression remaining) in the NCI-H460 sh0, but also correlates with the simultaneous direct or indirect regulation of a pool of different genes by Stau1 involved in the acquisition of the migratory phenotype (Figure 6A). This result confirmed that the effect of Stau1 depletion on cell migration is indeed partially mediated by THBS1. A similar result was observed for migration and invasion assays performed in the Boyden chambers (Supplementary Figure S5D,E).

Altogether, these results confirmed that the mRNA encoding THBS1 is a new target of Stau1 for Staufen mRNA-mediated decay. Furthermore, they suggest that the pro-metastatic properties of Staufen1 in lung cancer are at least partially mediated by THBS1.

## 3. Discussion

Stau1 is a pluripotent RBP responsible for the post-transcriptional regulation of a multitude of transcripts [9,10,77,78,79]. Consequently, even a modest modulation of its activity will affect numerous genes and pathways, potentially causing harmful defects to the cells [7]. Accordingly, from the meta-analysis of available databases, we highlight a clinical relevance of Stau1 expression for patients with non-small cell lung cancer. High Stau1 expression in tumor patients correlates with a better survival prognostic compared to low expression of Stau1. This advantage is also observed at the cellular level, as the lung tumor H460 cell line migrates, invades and metastasizes less efficiently than the Stau1-KD H460 cell lines. In addition, Stau1 expression reduces angiogenesis and facilitates the formation of mature blood vessels. These phenotypes are due to the misregulation of multiple transcripts coding for adhesion proteins, including CDH2 and THBS1 mRNA. THBS1 mRNA is a target of Stau1 binding and is post-transcriptionally degraded by SMD.

### 3.1. Levels of Stau1 Influence Tumor-Related Phenotypes of H460 Cancer Cells In Vitro

Stau1 depletion does not affect proliferation of H460 cells or the length of cell cycle G1, S and G2/M phases. This is consistent with previous studies in the colorectal cancer HCT116 cells and in the transformed HEK293 cells that showed that Stau1 depletion [21] or knock-out [74] did not impair cell proliferation. However, low expression of Stau1 protein promotes cell migration to ensure wound healing and facilitates migration in response to a gradient of chemotactic factors. Similarly, Stau1-depleted cells acquire invasive ability in Matrigel, suggesting that Stau1 depletion promotes extracellular matrix degradation and/or matrix–cell interactions to enable cell migration. Accordingly, defects were observed in spheroid formation of Stau1-KD cells revealing cell–cell adhesion defects. Our results indicate that a low expression of Stau1 favors cell interactions with the matrix to the detriment of interactions between cells, thus promoting the epithelial–mesenchymal transition, the acquisition of migratory ability and metastasis in vivo [76]. Altogether, these results indicate that the high Stau1 expression observed in lung cancer prevents cancer cells from acquiring migratory ability. In contrast, in embryonic subtypes of rhabdomyosarcoma, high Stau1 expression causes C-MYC translation via post-transcriptional gene regulation and results in significant effects on the proliferative and metastatic potential of these cells [80]. This is also observed in prostate cancer, where high levels of Stau1 regulate migration and invasion via the activation of focal adhesion kinase [81].

Consistently, Stau1 depletion affects the expression of a panel of genes involved in intercellular adhesion, cell adhesion to the extracellular matrix and EMT. Among those genes, THBS1 and CDH2 display a significant increase in the amounts of their mRNA and proteins. The functions of both genes are related to cell migration and EMT. CDH2 is a junction protein involved in extracellular matrix–cell interaction and favors migration and metastasis [82]. CDH2 promotes angiogenesis but also contributes to maintain the integrity of blood vessels by mediating pericytic–endothelial interactions [83,84,85]. CDH2 as an indicator of ongoing EMT and an increase of its expression has been correlated with the development of various types of carcinoma [86,87,88,89]. Similarly, THBS1 is a pluripotent protein previously described to play a role in tumor development, cell migration and angiogenesis [34,90,91,92,93,94,95,96,97,98,99,100]. THBS1 expression is generally enhanced in proliferating cells compared to quiescent cells. Originally discovered in platelets, THBS1 was then further characterized as a pluripotent effector involved in various regulons all disturbed in tumor cells. As a result, THBS1 plays important roles in carcinogenesis. However, its functions in angiogenesis and tumor progression remain controversial. Indeed, THBS1 is an inhibitor of both processes in many cancers, while, in others, it acts as a positive regulator. It is plausible that the role of THBS1 in tumors depends on the cell-specific factors and signaling pathways that are activated in each specific tumor and contributes to optimize cell growth and survival [101]. One of these factors could be Stau1, whose expression controls the amounts of THBS1 via SMD. A defect in Stau1 expression impacts the migratory ability of cells and by extension, could lead to the acquisition of an aggressive phenotype in vivo. 

### 3.2. Stau1 Favors the Maturation of Efficient Blood Vessels

In this paper, we demonstrate that Stau1 expression in the lung non-small cancer cells H460 cell line prevents the acquisition of a pro-metastatic phenotype and favors a mature vasculature instead of promoting angiogenesis and the neo-genesis of immature and permeable blood vessels. Both phenotypes are tightly related since maturation of blood vessels with pericyte coverage ensures proper perfusion of cancer cells and reduces stresses induced by oxygen and nutriments deprivation compared to immature tumor blood vessels [76]. Because of defects in permeability, immature blood vessels fail to carry essential components to sustain cancer cells and favor the establishment of an environmental pressure of selection due to stresses. Consequently, this context favors the selection of resistant cells and promotes EMT, thus leading to an increase of metastasis. The presence of mature vasculature in H460 xerographs is consistent with the reduced metastatic phenotype observed in these mice compared to those injected with Stau1-KD H460 cells. In addition, the anti-metastatic phenotype is consistent with the meta-analysis of patients with non-small cell lung cancer that links high expression of Stau1 to a better clinical outcome (KMplot). These phenotypes are likely due to the misregulation of multiple transcripts coding for adhesion molecules and especially to the downregulation of THBS1 mRNA via SMD and protein in tumor cells. Our results in lung cancer are opposite to those in glioma where increased SMD was associated with enhanced angiogenesis, proliferation and cell migration [69,70]. In vascular endothelial cells derived from glioma, enhanced SMD stabilizes the blood-tumor barrier and reduced its permeability [71].

### 3.3. Stau1 Controls a Novel RNA Regulon Involving the THBS1 mRNA 

Stau1 is well known for its role in the post-transcriptional regulation of mRNA regulons. In non-transformed cells, the Stau1/E2F1 positive loop regulates cell cycle phase transition and cell proliferation (see above). Upon cell transformation, other loops develop that control cell proliferation, migration and invasiveness as well as angiogenesis. Notably, enhanced expression of long non-coding RNAs modifies Stau1-mediated mRNA decay and changes cell properties. In glioma-derived endothelial cells, Stau1 binds to the ZNF655 mRNA coding for a zinc finger protein following enhanced expression of the lncRNA LINC00346 and thus enhances its degradation via enhanced SMD. ZNF655 is an inhibitory transcription factor targeting the promoter region of the ankyrin repeat and KH domain containing protein 1 (ANKHD1) gene. Interestingly, upregulated ANKHD1 binds LINC00346 lncRNA and promotes its stability, thus forming the Stau1-regulated ANKHD1/LINC00346/ZNF655 feedback loop. In addition, ANKHD1 plays a role in the regulation of the cell cycle and cell cycle progression [102,103]. LINC00346 has been shown to be oncogenic [104]. Similarly, in glioma, SMD is enhanced due to the upregulation of the lncRNA HCG15 that is stabilizes by the binding of PABPC5. Enhanced SMD reduces the amount of the inhibitory transcription factor ZNF331 and consequently upregulates the transcription of LAMC2 and PABPC5, forming the Stau1-regulated PABPC5/HCG15/ZNF331 positive loop [70].

We now show that Stau1 controls other regulons of mRNAs coding for cell adhesion molecules. Notably, Stau1 binds an intramolecular double-stranded stem in the 3′UTR of THBS1 mRNA and degrades this mRNA via SMD. Interestingly, the low amount of THBS1 in non-small cell lung cancer cells has no effect on cell proliferation but influences cell migration, invasiveness and metastasis. The discrepancy concerning the opposite functions of THBS1 regulation of angiogenesis, proliferation and migration reported in the literature highlights the importance of the concomitant regulation of genes involved in common pathways and the presence or not of several co-factors that could be expressed in a cell-specific way. The different observed functions of THBS1 may not only be due to the level of expression of THBS1 in different contexts but more likely to a balance established in the cell between co-factors acting altogether to regulate a common pathway under the control of Stau1. In addition, Stau1 was shown to also directly bind THBS4 mRNA in HEK293 cells, another member of the Thrombospondin family [10]. Subsequently, a defect in Stau1 expression directly affects several regulons to change cell properties. Several studies support these observations by describing how Stau1 directly regulates its targets, but also more broadly entire signaling pathways [22,32,52,65,67,68,70,71]. Therefore, the modification of a single RBP such as Stau1 impacts the expression of many downstream targets, but also all the signaling pathways in which these genes are involved. Moreover, other RBPs such as the human antigen R protein (HuR) [105,106,107], the nucleolar protein 7 (NOL7) [108] and the AU-rich element RNA-binding protein 1 (AUF1) [109] can bind and regulate THBS1 mRNA. Thus, the observed phenotypes subsequent to Stau1 modulation are not due to the defect of regulation of one direct target but rather to the addition of subtle gene modulations acting together in a complex network and leading to establishment of a new balance of pro- and anti-factors involved in migration, angiogenesis and proliferation, thus resulting in the acquisition of a new phenotype.

## 4. Material and Method

### 4.1. Database Retrieval

The prognostic value of Stau1 mRNA expression in lung cancer was assessed in the Kaplan–Meier plotter database [110]. The Stau1 gene data were uploaded into the database according to the median expression level. Patient samples were divided into two groups corresponding to high vs. low Stau1 mRNA expression, overall survival (OS) and progression-free survival (PFS) were investigated the by a Kaplan–Meier survival plot. The hazard ratios (HRs) with 95% confidence intervals (CIs) and the log-rank *p*-values were calculated automatically on the webpage. A *p*-value of less than 0.05 was considered statistically significant. The expression profiles of Stau1 genes in non-small cell lung carcinoma and adjacent normal lung tissues were analyzed using the OncomineTM [111] database (Compendia Bioscience, Ann Arbor, MI, USA). The meta-analysis was performed using the Hou study data [72]. 

### 4.2. Western Blot Analysis and Antibodies 

Total cell extracts were prepared in lysis buffer (50 mM Tris–HCl pH 7.4 (MilliporeSigma, St. Louis, MO, USA)), 1 mM EDTA (MilliporeSigma, St. Louis, M0, USA), 1 mM EGTA (MilliporeSigma, St. Louis, MO, USA), 0.5% NA-deoxycholate (MilliporeSigma, St. Louis, MO, USA), 1% NP40/Tergitol (Merck, Darmstadt, Germany) 10% glycerol (Merck, Darmstadt, Germany), 0.1% SDS (MilliporeSigma, St. Louis, MO, USA), 150 mM NaCl (MilliporeSigma, St. Louis, MO, USA), 1 mM dithiothreitol (DTT) (MilliporeSigma, St. Louis, MO, USA) and a protease inhibitor cocktail (Roche, Basel, Switzerland), and protein concentrations were determined by Bradford assays. Cell extracts (15 to 30 μg) were analyzed by Western blotting using nitrocellulose membrane. Data were collected either on X-ray films (Fujifilm, Tokyo, Japan) or with the ChemiDoc MP Imaging System (Bio-Rad Laboratories, Hercules, CA, USA).

Incubations with primary antibodies were performed overnight at 4 °C prior to incubation with HRP-conjugated secondary antibody polyclonal anti-mouse (1/3000) P0447; polyclonal anti-rabbit (1/5000) (P0448, Agilent, Santa Clara, CA, USA) for 1 to 3 h at room temperature. Primary rabbit polyclonal antibodies against Stau1 (A303-956A, Bethyl, Montgomery, AL, USA, 1:1000), THBS1 (18304-1-AP, Proteintech, Manchester, UK, 1:500), PCNA (P12004, Bethyl, Montgomery, AL, USA 1:1000), mouse monoclonal anti-CDH2 (D4R1H Cell Signaling Technology, Danvers, U.S.A., 1:1000), hnRNP U (3G6 Cell Signaling Technology, Danvers, MA, USA, 1:1000) and mouse monoclonal anti-β-Actin (A5441 MilliporeSigma, St. Louis, MO, USA, dilution 1:1000) were used to perform Western blot analysis. Monoclonal anti-mouse (A9917, Merck, Darmstadt, Germany 1:3000) or rabbit (A0545, Merck, Darmstadt, Germany, 1:5000) secondary antibodies were then used for 3 h incubation.

### 4.3. Human Tissue Samples and Protein Extracts

We prospectively recorded patients with clinico-pathological data, who underwent surgery for lung carcinoma at Rangueil Hospital (Toulouse, France) from 2007 to 2008. Tissues samples (tumoral tissues and corresponding non-tumoral counterpart) were obtained from pulmonary lobectomy or pneumonectomy specimens during surgery and were immediately frozen in liquid nitrogen and stored at −80 °C. The protocol had local ethical committee approval. Consents were obtained from patients before surgery. 

Frozen tissue samples were pulverized with “Mikro-Dismembrator” (Sartorius, Aubagne, France) and resuspended in lysis buffer and Western blot was performed as described in the above section. 

Human Lung Cancer microarray LC721 and LC722 (US Biomax, Rockville, MD, USA) was processed according to the supplier’s recommendations.

### 4.4. Cell Culture

NCI-H460 (HTB-177, ATCC, Manassas, VA, USA) cells were cultured in RPMI-1640 Medium (Bio-Rad Laboratories, Hercules, CA, USA) supplemented with 10% fetal bovine serum (Thermo Fisher, Waltham, MA, USA), 100 μg/mL streptomycin and 100 units/mL penicillin (Thermo Fisher, Waltham, MA, USA), under 5% CO_2_ atmosphere. Cells were tested for mycoplasma contamination using the Mycoalert mycoplasma detection kit (Lonza LT07-218, Lonza, Basel, Switzerland) according to the manufacturer’s instructions.

### 4.5. Generation of Stable Stau1-KD Cell Lines 

Lentiviral constructs targeting Stau1 (TRCN0000158514, corresponding to the sequence CCGGCAAGTGTTTGAGATTGCACTTCTCGAGAAGTGCAATCTCAAACACTTGTTTTTTG-sh1; and TRCN0000159875, corresponding to the sequence CCGGGTGTTTGAGATTGCACTTAAACTCGAGTTTAAGTGCAATCTCAAACACTTTTTTG-sh2) and non-targeting shRNA vectors (SHC002V, shScr) were purchased from Merck, Darmstadt, Germany, and produced with 293T (CRL-3216, ATCC, Manassas, VA, USA) cells cotransfected with the two helper plasmids pLvVSVg and pLvPack (MilliporeSigma, St. Louis, MO, USA). After transduction, NCI-H460 cells were selected and maintained using 2.5 μg/mL puromycin (MilliporeSigma, St. Louis, MO, USA). Gene silencing was confirmed by quantitative RT-PCR (RT-qPCR) and Western blot analysis.

### 4.6. siRNA and DNA Transfections

THBS1 expression was knock-down in NCI-H460 using a pool consisting of an equimolar mix of 4 siRNAs 5′GGACUGCGUUGGUGAUGUA3′, 5′CCAAAGACGGGUUUCAUUA3′, 5′CGAUGACUAUGCUGGAUUU3′, 5′CCACGAGGGCUCAGGGAUA3′ (Eurofins Genomics, France). UPF1 expression was knock-down in NCI-H460 using a siRNA targeting the sequence 5′-r(GAUGCAGUUCCGCUCCAUU)d(TT)-3′ [17] and compared to a non-targeting siRNA control, siSCR 5′-r(GAAUAUGUUCGUAAUCACU)d(TT)-3′ siRNAs were transfected when cells reached approximately 40–60% confluency using INTERFERin^®^ reagent (Polyplus-transfection, Illkirch, France) according to the manufacturer’s instructions. For DNA plasmids, transfections were performed using JetPEI^®^ (Polyplus-transfection, Illkirch, France) when cells reached approximately 50–60% confluency according to the manufacturer’s instructions. Cells were harvested 48 h post-transfection with plasmids or siRNAs to prepare total RNA and/or protein extracts.

### 4.7. Plasmids and Cloning Strategies

The plasmid containing the Firefly luciferase reporter gene under the control of the THBS1 promoter region and the one allowing THBS1 overexpression and the plasmids coding for Stau1^55^-FLAG_3_ were generous gifts from F. Cabon and L. DesGroseillers Labs, respectively. Control peporter vector pRL-CMV was from Promega Corporation, Madison, Wisconsin, USA. Stau1^55^-FLAG_3_ plasmid was mutagenized in order to restore its expression in the cell lines expressing sh1 and sh2 shRNAs directed against the RNA encoding Stau1. For this purpose, silent mutations were introduced in the sequence so as to change the GTGTTTGAGATTGCACTTAAA sequence (GlnValPheGluIleAlaLeuLys) by GTCTTCGAAATCGCCTTGAAG using the following oligonucleotides 5’-CTTCGAAATCGCCTTGAAGCGGAACTTGCCTGTGAATTT-3’ and 5’-CTTCAAGGCGATTTCGAAGACTTGACTTATTTCAGATTTATTGAG-3’. Mutagenesis was performed with a Quickchange kit according to the manufacturer recommendations (Agilent, Santa Clara, CA, USA). 

The constructions of THBS1 3′UTR deletion (Figure 7B), DNAs corresponding to the full-length THBS1 (FL) 3′UTR and the four deletions named Δ1, Δ2, Δ3 and Δ4 were PCR-amplified from NCI-H460 total cDNAs using Phusion polymerase (Thermo Fisher, Waltham, MA, USA) and oligonucleotide primers described in Table 1. Constructions were then cloned into pSCT40 vector. Constructions allowing the isolation of the two candidates’ stem loop structures were generated using the full-length THBS1 3′UTR previously obtained as a template and the primers listed Table 2.

### 4.8. Total mRNA Extracts Purification, RT-qPCR and RT-ddPCR Analysis

mRNA extracts were purified using TriReagent^®^ (MilliporeSigma, St. Louis, MO, USA) according to the manufacturer’s protocol and purity of each extract was assessed using Nanodrop. Extracts were stored at −80 °C. Prior to performing RT-qPCR, 1 µg of each sample was treated with DNaseI (18068015, Thermo Fisher Scientific, Waltham, MA, USA) for min. DNaseI was inactivated by adding EDTA at 65 °C for 10 min. Reverse transcription was performed using RevertAids^TM^ kit (Thermo Fisher Scientific, Waltham, MA, USA) and random hexamer according to the manufacturer’s protocol, and cDNAs produced were conserved at −20 °C. cDNAs were amplified by RT-qPCR after 30 cycles using StepOne™. RT-ddPCR was performed using Evagreen soFast reagent according to the manufacturer’s protocol (Bio-Rad, Hercules, CA, USA). HPRT gene was used for results normalization. Table 3 provides the sequences of the primers used to amplify the corresponding genes.

### 4.9. RNA Immunoprecipitation and RNA Chromatography Assays

RNA immunoprecipitations were performed using the RiboCluster ProfilerTM (MBL, Nagoya, Japan) kit according to the manufacturer’s protocol. Cells were transfected with the appropriated plasmid Stau1^55^-FLAG_3_ and lysed 48 h later with 1 mL of lysis buffer provided by the kit. Inputs were prepared with 10 µL of each lysed sample. EZviewTM (E3403, MilliporeSigma, St. Louis, MO, USA) beads were pre-coated with 15 µg of anti-Flag (F3165, MilliporeSigma, St. Louis, MO, USA) antibody and incubated for one hour at room temperature. Precipitated RNAs were then analyzed by RT-qPCR. RNA chromatography was performed as described in Lamaa et al. [112].

### 4.10. Analysis of THBS1 mRNA Half-Life and Stability upon Stau1 Expression

NCI-H460 cell lines WT expressing sh0 or siSCR, Stau1-KD cell lines sh1 or sh2 and Upf1-KD cell line were treated with 10 μg/mL Actinomycin D (EU0450, Euromedex, Souffelweyersheim, France) (t = 0 h). Cells were collected at different time points (0 h, 1 h, 6 h, 9 h) and total mRNA extracts were prepared. 

NCI-H460 control and Stau1-KD cells were treated or untreated with 10 μg/mL actinomycin D (t = 0 h). Cells were then harvested 3 h, 6 h, and 9 h later. Total mRNAs were extracted, and THBS1 mRNA expression was analyzed by RT-qPCR and normalized with the negative SMD activity control gene HPRT

### 4.11. Luciferase Reporter Assays

Luciferase assays were performed with a dual-luciferase Reporter Assay System (E1960, Promega Corporation, Madison, WI, USA) according to the manufacturer protocol on 50 mL of cell lysate. Luciferase activity was recorded with a Centro xs3 (Berthold Technologies, Thoiry, France) by using 2–5 sec integration time.

### 4.12. Migration, Invasion and Proliferation Assays

For the migration assays, cells were harvested and resuspended in 0.5% RPMI 1640 medium, and 1 × 10^5^ cells were placed into Boyden upper chambers (ECM 508, Merck, Darmstadt, Germany) containing an 8.0 µm pore membrane. For the invasion assays, 1 × 10^5^ cells were placed into upper chambers coated with 250 µL of Matrigel (356234, Corning/Thermo Fisher, Waltham, MA, USA). The chambers were subsequently inserted into the wells of a 24-well plate and incubated for 24 h in RPMI 1640 medium with 10% FBS. The cells remaining on the upper surface of the membranes were removed, whereas the cells adhering to the lower surface were fixed with 4% paraformaldehyde (MilliporeSigma, St. Louis, MO, USA), stained in a dye solution containing 0.05% crystal violet and 25% methanol, and counted under a microscope (TS100, Nikon, Tokyo, Japan). Five random fields were analyzed on each insert and the results were averaged among 3 independent experiments. Wound healing assays were performed by seeding cells until they reached 80 to 90% confluency. A scratch was performed, and pictures were taken at t = 0 h and 16 h. Quantification of the wound area was analyzed with Fiji-ImageJ freeware. Proliferation was assessed by performing a growth curve. Cells were plated at the same density (day = 0) and harvested every day for 6 days. The number of cells was counted with a Coulter (Beckman, Pasadena, CA, USA).

### 4.13. Soft Agar Assay for Anchorage-Independent Growth and Spheroid Formation Assays

To evaluate the effect of Stau1 depletion on in vitro tumorigenic potential of NCI-H460 cells, colony formation assay on soft agar was carried out. For this anchorage-independent growth assay, NCI-H460 was seeded between two layers of culture medium containing soft agar. The base layer was prepared with 0.5% agar and plated on 35 mm dishes and the top layer mixed with cells (5000 cells) was prepared with 0.35% agar as reported in Armstrong et al. [113]. An amount of 3 mL of culture medium was added and renewed every three days. Plates were incubated at 37 °C in an incubator with 5% CO_2_ and observed daily for colony formation by microscope observation until the soft agar colonies reached visible size. The soft agar-grown colonies were stained with PBS and colored with 0.5% crystal violet in 50% methanol for 10 min and washed three times with a PBS solution before counting on day 14. The number of colonies in each treatment groups was counted.

To evaluate the ability of NCI-H460 WT and Stau1-KD to form 3D spheroids, cells were harvested using trypsin-EDTA, and plated at a density of 30,000 cells per well in low-binding plates (Nunclon™ Sphera™ 96-Well, Thermo Fisher, Waltham, MA, USA). Spheroids were considered fully formed after 4 days in vitro [26]. Images of spheroids were taken with microscope (TS100, Nikon, Tokyo, Japan) at day 4, 8 and 20 to monitor spheroid formation. 

### 4.14. In Vivo Metastasis Assays

The nude mice were obtained from Janvier-Labs (Le-Genest-Saint-Isle, France). An amount of 1 × 10^6^ NCI-H460 cells expressing the different shRNAs were used to perform subcutaneous xenografts to nude mice (10/group, female nu/nu). The volume of each tumor was monitored from day 7 to day 35 as indicated in Figure 4A. The mice were euthanized at day 35 and tumors, lungs and livers were collected for subsequent analysis. Half of the tumors were embedded in optimal cutting temperature (OCT) compound (Tissue Tek, Sakura Europe, France) and sections were generated with a CM1950 Cryostat (Leica, Wetzlar, Germany) on glass coverslips. Total RNA and protein extracts were isolated with the remaining tumoral tissue. RNA extraction was performed on lungs and liver with TriReagent (T9424, Merck, Darmstadt, Germany) and further analyzed by RT-qPCR.

### 4.15. Flow-Cytometry Analysis

Cells were collected, washed with PBS and fixed overnight at 4 °C in 70% Ethanol, diluted in PBS. The next day, cells were washed with PBS and incubated for 30 min in PBS with 0.1% Triton X-100, RNaseA (0.2 mg/mL) CL-APA297HU01-10UG (Euromedex, Souffelweyersheim, France) and propidium iodide (20 mg/mL) SE-S6874-50MG (Euromedex, Souffelweyersheim, France) at room temperature. The protocol was adapted from Darzynkiewicz et al., in *Current Protocols in Cell Biology* [114]. Stained cells were analyzed on a LSRII flow cytometer (BD Biosciences, Franklin Lakes, NJ, USA) with exclusions of doublets. Analysis of the results was performed with FlowJo software.

### 4.16. Epifluorescence Microscopy and Immunohistochemistry

Immunohistochemistry on patient tumor samples was performed according to Hofman and Taylor in *Current Protocols in Immunology* [115]. Anti Stau1 (ab73478, Abcam, Waltham, MA, USA) antibody was used at a 1/200 dilution. Secondary antibody (PI-1000-1, Vector Laboratories, CA, USA) was diluted to 1/1000.

To analyze the expression of THBS1 and blood vessels markers, coverslips were rehydrated for 30 min at room temperature, then fixed in formalin for 20 min at 4 °C. PBS 1X/BSA 1%/Triton 0.5% was used to perform permeabilization and blockage for 45 min. Cells were then stained with anti-THBS1 (MA5-13398, Thermo Fisher, Waltham, MA, USA), anti-αSMA (AB7817, Abcam, Cambridge, UK) or anti-CD31 (553370, Beckton Dickinson, Franklin Lakes, NJ, USA) (dilution 1:200) for 1 h at room temperature. Secondary antibody anti-mouse 546 and anti-rabbit 488 (A11060 and A21467, Invitrogen, Carlsbad, CA, USA) were added for 1 h at room temperature. Coverslips were washed once with PBS and incubated for 10 min with PBS containing 0.5 μg/mL DAPI (ref. 1050A, Euromedex, Souffelweyersheim, France) at room temperature. Coverslips were mounted using Dako mounting medium (ref. 83023, Dako/Agilent, Santa Clara, CA, USA). Images were acquired with a Leica DMi8 microscope (Leica, Wetzlar, Germany). Images processing was performed using Molecular Devices or Fiji freeware [116]. 

## Figures and Tables

**Table 1:** HBS1 3′UTR deletions.

Primer	Sequence
THBS1 3′UTR forward	5′-GTAATACGACTCACTATAGGGGCAGTCTAGAGTCGGGGCGG-3′
THBS1 FL reverse	5′-TTTTTTTTTTTTTTTTTTTTTTTTTTTTTTACAAGGAACAACAATAAATCATATGG-3′
Δ1 reverse	5′-TTTTTTTTTTTTTTTTTTTTTTTTTTTTTTCCAGAAGTCAGATGCTCAAGGGGC-3′
Δ2 reverse	5′-TTTTTTTTTTTTTTTTTTTTTTTTTTTTTTCCAACAATTCTTCAATTCAGTGTGC-3′
Δ3 reverse	5′-TTTTTTTTTTTTTTTTTTTTTTTTTTTTTTTGCACTGCCTTACACTGGTTTG-3′
Δ4 reverse	5′-TTTTTTTTTTTTTTTTTTTTTTTTTTTTTTGCCCTCCCCTTAGTGCTTTG-3′

**Table 2:** Constructions 3′UTR primers.

Constructions	Primer	Sequence
a	forward	5′-GTAATACGACTCACTATAGGGGCAGTCTAGAGTCGGGGCGG-3′
	reverse	5’-GCACTGCCTTACACTGGTTTG-3′
b	forward	5′-AAAGTAATACGACTCACTATAGGGCAAACCAGTGTAAGGCAGTGC-3′
	reverse	5′-TTTTTTTTTTTTTTTTTTTTTTTTTTTTTTACAAGGAACAACAATAAATCATATGG-3′
c	forward	5′-AAAGTAATACGACTCACTATAGGGCAAACCAGTGTAAGGCAGTGC-3′
	reverse	5′-GAGCACAAGGGGCAGAGCAG-3′
d	forward	5′-AAAGTAATACGACTCACTATAGGGCTGCTCTGCCCCTTGTGCTC-3′
	reverse	5′-TTTTTTTTTTTTTTTTTTTTTTTTTTTTTTACAAGGAACAACAATAAATCATATGG-3′
e	forward	5′-AAAGTAATACGACTCACTATAGGGCAAACCAGTGTAAGGCAGTGC-3′
	reverse	5′-GGTTGATAATAATTTTGTGCCATTGT-3′
f	forward	5′-AAAGTAATACGACTCACTATAGGGACAATGGCACAAAATTATTATCAACC-3′
	reverse	5′-GAGCACAAGGGGCAGAGCAG-3′

**Table 3:** RT-qPCR primers.

Gene ID	Primer	Sequence
HPRT	forward	5′-TGCTTTCCTTGGTCAGGCAGT-3′
	reverse	5′-CTTCGTGGGGTCCTTTTCACC-3′
Stau1	forward	5′-GATCCTGCAGAATGAGCCCC-3′
	reverse	5′-CACCTCGAAATTCACAGGCAA-3′
CDH2	forward	5′-GATCCTGCAGAATGAGCCCC-3′
	reverse	5′-CACCTCGAAATTCACAGGCAA-3′
THBS1	forward	5′-GGGGCGTCAATGACAATTTCCAG-3′
	reverse	5′-TCACCACGTTGTTGTCAAGGGT-3′
TWIST1	forward	5′-GCAGGGCCGGAGACCTA-3′
	reverse	5′-TTGGATTTTGCTCTTCTAATTTCCA-3′
SNAI2 (SLUG)	forward	5′-GCGGCAAGGCGTTTTCCAGA-3′
	reverse	5′-GCAGTGAGGGCAAGAAAAAGGC-3′
VIM (Vimentin)	forward	5′-CATGCGCCTCCGGGAGAAAT-3′
	reverse	5′-TCAAGACGTGCCAGAGACG-3′
ZEB1	forward	5′-TAAGCGCAGAAAGCAGGCGA-3′
	reverse	5′-ACAGTCAGCTGCATCTGTAACACT-3′
SNAI1	forward	5′-AGTGCCTCGACCACTATGCC-3′
	reverse	5′-TCGTAGGGCTGCTGGAAGGTA-3′
ZEB2	forward	5′-GCCATCTGATCCGCTCTTATC-3′
	reverse	5′-ACCTGTGTCCACTACATTGTC-3′
SERPINE	forward	5′-GTGGACTTTTCAGAGGTGGAG-3′
	reverse	5′-GAAGTAGAGGGCATTCACCAG-3′
KRT19	forward	5′-GCGAGCTAGAGGTGAAGATC-3′
	reverse	5′-AATCCTGGAGTTCTCAATGGTG-3′
CAV2	forward	5′-TCAACTCGCATCTCAAGCTG-3′
	reverse	5′-GATTTCAAAGAGGGCATGGC-3′
CALD1	forward	5′-TGTGGGAGAAAGGGAATGTG-3′
	reverse	5′-AAGGTTTGGGAGCAGGTG-3′
NOTCH1	forward	5′-TGCCTGGACAAGATCAATGAG-3′
	reverse	5′-CAGGTGTAAGTGTTGGGTCC-3′
MET	forward	5′-GCCCAAACCATTTCAACTGAG-3′
	reverse	5′-ACCTGTTATTGTGCTCCCAC-3′
EGFR	forward	5′-AAGCCATATGACGGAATCCC-3′
	reverse	5′-GGAACTTTGGGCGACTATCTG-3′
SMAD2	forward	5′-GATCCTAACAGAACTTCCGCC-3′
	reverse	5′-CACTTGTTTCTCCATCTTCACTG-3′
BMP1	forward	5′-CTCCCCTGAATACCCCAATG-3′
	reverse	5′-ACCTCCACATAGTCGTACCAG-3′
PLEK2	forward	5′-ACTGTGGAGTTAAGTGGCAC-3′
	reverse	5′-GGAAGGGTCATAGTAATGCAGG-3′
SNAI1	forward	5′-GGAAGCCTAACTACAGCGAG-3′
	reverse	5′-CAGAGTCCCAGATGAGCATTG-3′
MSN	forward	5′-TCGCAAGCCTGATACCATTG-3′
	reverse	5′-TTCTCTTTCTCCTTCTCTGCC-3′
NUDT13	forward	5′-CCTCTTTCATAGTCTGGCTCC-3′
	reverse	5′-GCATCCAATCAGCACAGAATC-3′
STAU2	forward	5′-ATCTACGCTTCCCAAACCAG-3′
	reverse	5′-GAATGGCTTTGGATCTAATGGC-3′
DESM1	forward	5′-GATCAATCTCCCCATCCAGAC-3′
	reverse	5′-GACCTCAGAACCCCTTTGC-3′
ITGAV	forward	5′-AGAATCAAGGAGAAGGTGCC-3′
	reverse	5′-GGCGAGTTTGGTTTTCTGTC-3′
ILK	forward	5′-CAAACACTCTGGCATTGACTTC-3′
	reverse	5′-CTGCTCTTCCTTGTACTCCAG-3′
IL1RN	forward	5′-CCTCATGCTCTGTTCTTGGG-3′
	reverse	5′-TGTCCTGCTTTCTGTTCTCG-3′
VCAN	forward	5′-CAGTCATAGCAACTCCAGAGC-3′
	reverse	5′-CTCCTGCCTTTCCCATCTTATC-3′
MMP2	forward	5′-ACCCATTTACACCTACACCAAG-3′
	reverse	5′-TGTTTGCAGATCTCAGGAGTG-3′
ITGA5	forward	5′-ATACTCTGTGGCTGTTGGTG-3′
	reverse	5′-CTGTTCCCCTGAGAAGTTGTAG-3′
STAT3	forward	5′-TTCTGGGCACAAACACAAAAG-3′
	reverse	5′-TCAGTCACAATCAGGGAAGC-3′
MST1R	forward	5′-ATGTGCTGATTCCCCATGAG-3′
	reverse	5′-TGCGACTTAGTGACTTGATGG-3′
JAG1	forward	5′-GGACTATGAGGGCAAGAACTG-3′
	reverse	5′-AAATATACCGCACCCCTTCAG-3′
FGFBP1	forward	5′-ACCCAGATATGGCAAACCAG-3′
	reverse	5′-ACCCGTTCTCTTTTGACCTC-3′
OCLN	forward	5′-GCAAAGTGAATGACAAGCGG-3′
	reverse	5′-CACAGGCGAAGTTAATGGAAG-3′
TIMP1	forward	5′-TTCTGCAATTCCGACCTCG-3′
	reverse	5′-TCATAACGCTGGTATAAGGTGG-3′
DSP	forward	5′-ACCAGAACCAGAACACCATC-3′
	reverse	5′-GGGCAAAACACTCATCCAATTC-3′
SPARC	forward	5′-CGACTCTTCCTGCCACTTC-3′
	reverse	5′-GGAATTCGGTCAGCTCAGAG-3′
IGFBP	forward	5′-CACAGGAGACATCAGGAGAAG-3′
	reverse	5′-GATCCTCTTCCCATTCCAAGG-3′
Pac	forward	5′-GCTCGACATCGGCAAGGTGT-3′
	reverse	5′-GAACCGCTCAACTCGGCCAT-3′
GAPDH	forward	5′-TCAAGGCTGAGAACGGGAAG-3′
	reverse	5′-CGCCCCACTTGATTTTGGAG-3′
CDH1	forward	5′-CCCAATACATCTCCCTTCACAG-3′
	reverse	5′-CCACCTCTAAGGCCATCTTTG-3′
ANKRD57	forward	5′-AGGAACGACCTGTTAAAGGC-3′
	reverse	5′-TTCTGGTCTCACTTCCTTACAAC-3′

## Data Availability

The data presented in this study are available on request from the corresponding author.

## Supplementary Materials

The following are available online at https://www.mdpi.com/article/10.3390/ijms23010215/s1.
