# Supplementary figures and images for: High Level of Staufen1 Expression Confers Longer Recurrence Free Survival to Non-Small Cell Lung Cancer Patients by Promoting THBS1 mRNA Degradation
Source: Int J Mol Sci. 2021 Dec 25;23(1):215. doi: 10.3390/ijms23010215 (PMC8745428; doi:10.3390/ijms23010215)

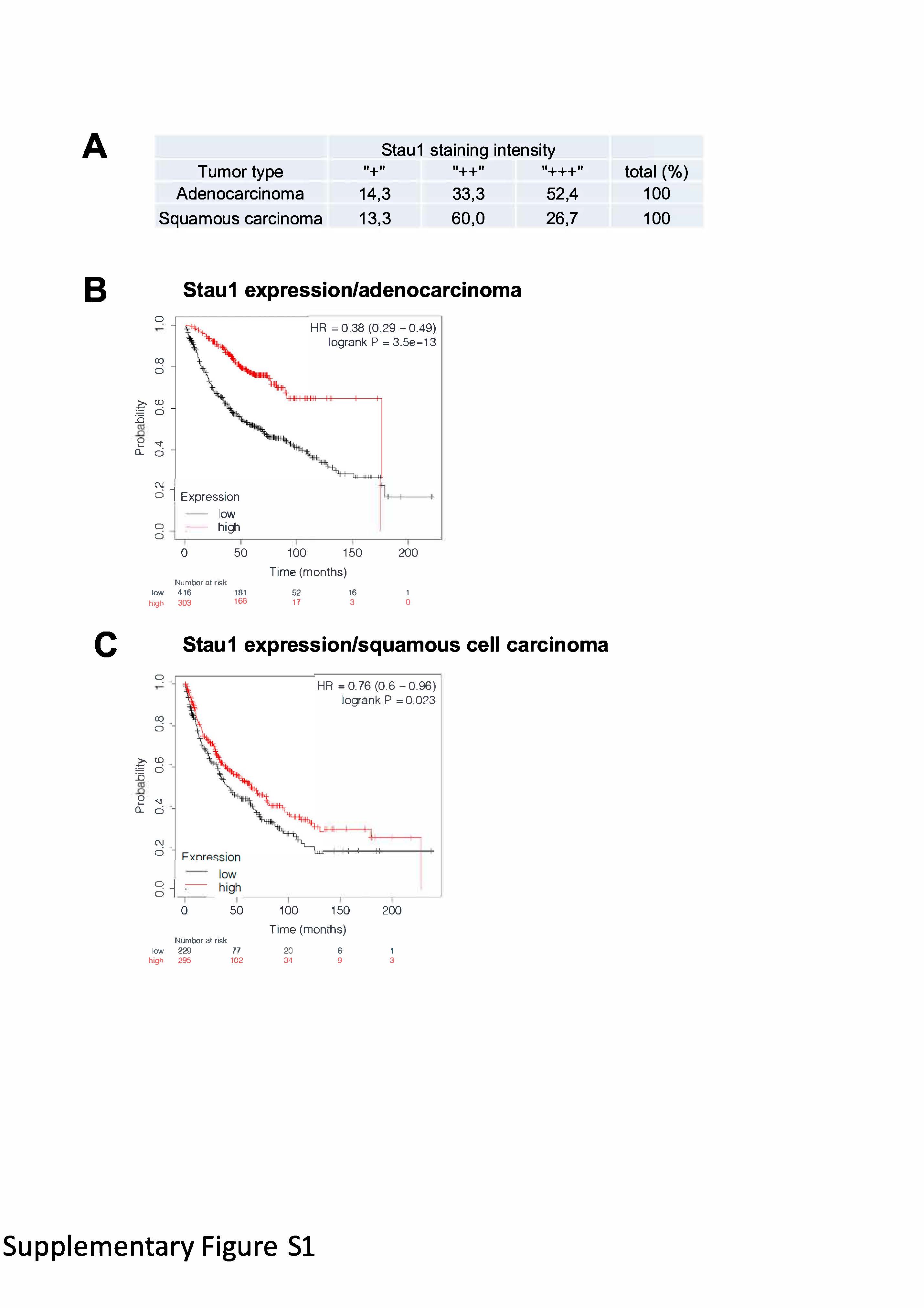

Supplement: Supplementary file 1 [file ijms-23-00215-s001.zip › SupFig1.jpg]

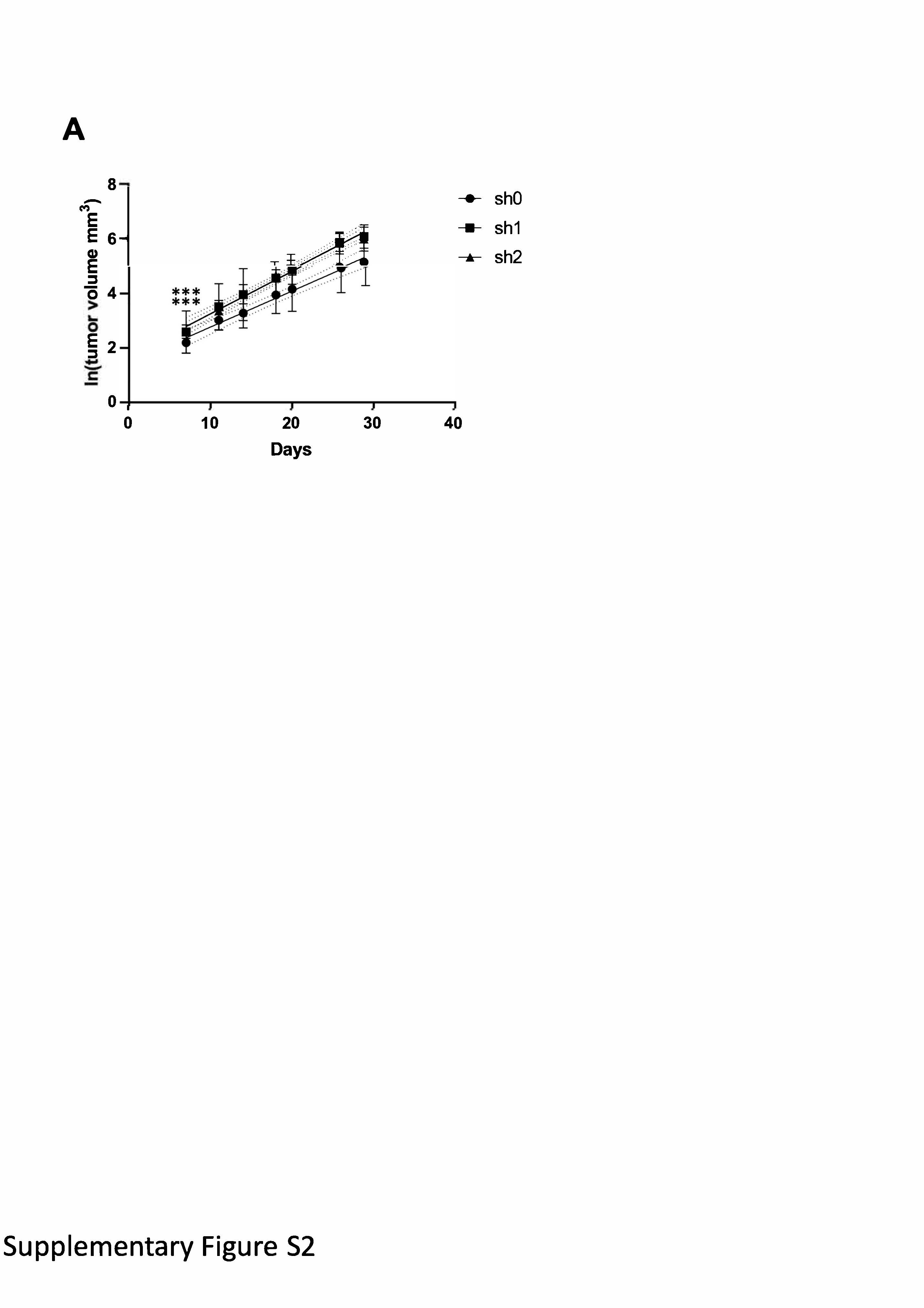

Supplement: Supplementary file 1 [file ijms-23-00215-s001.zip › SupFig2.jpg]

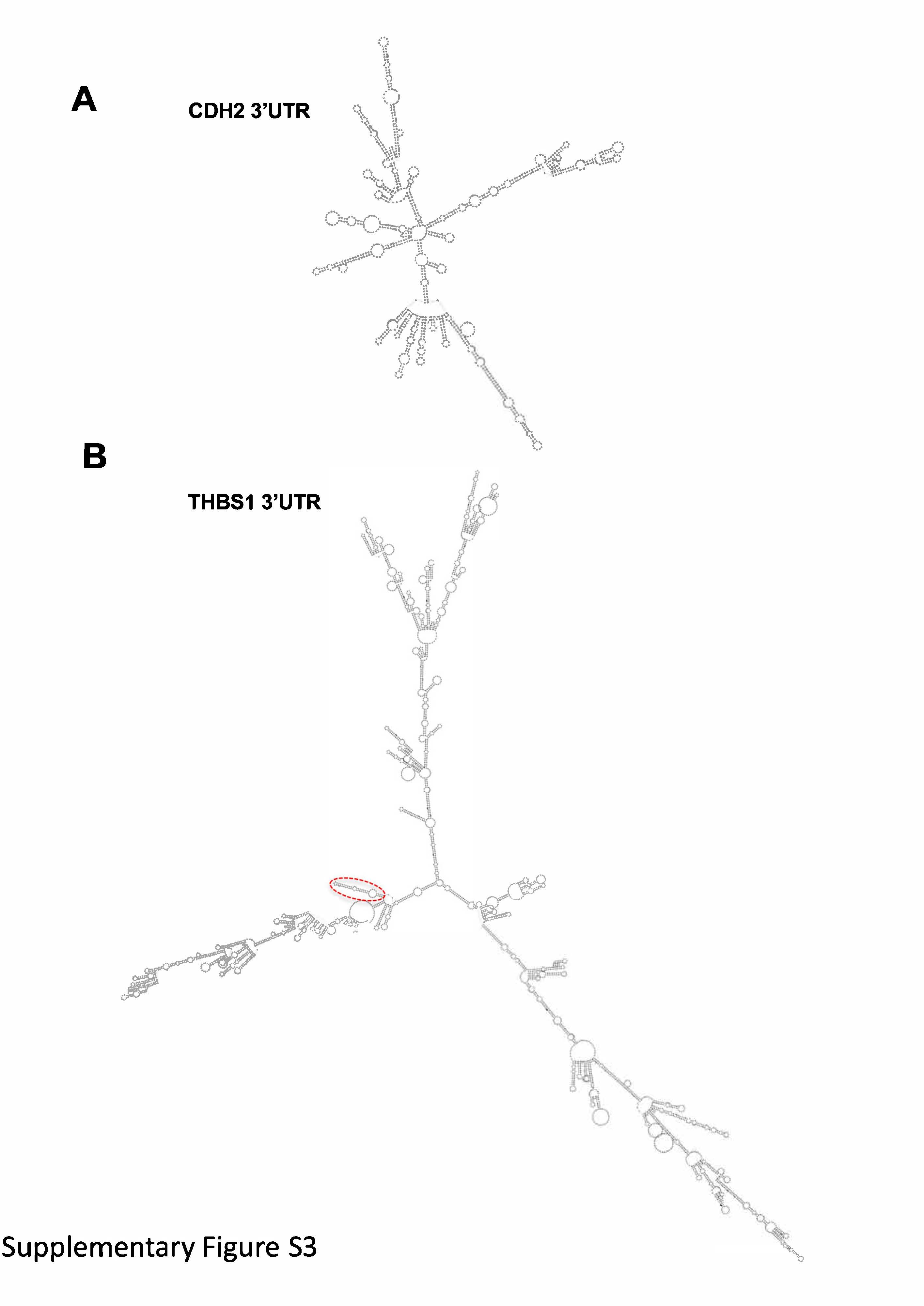

Supplement: Supplementary file 1 [file ijms-23-00215-s001.zip › SupFig3.jpg]

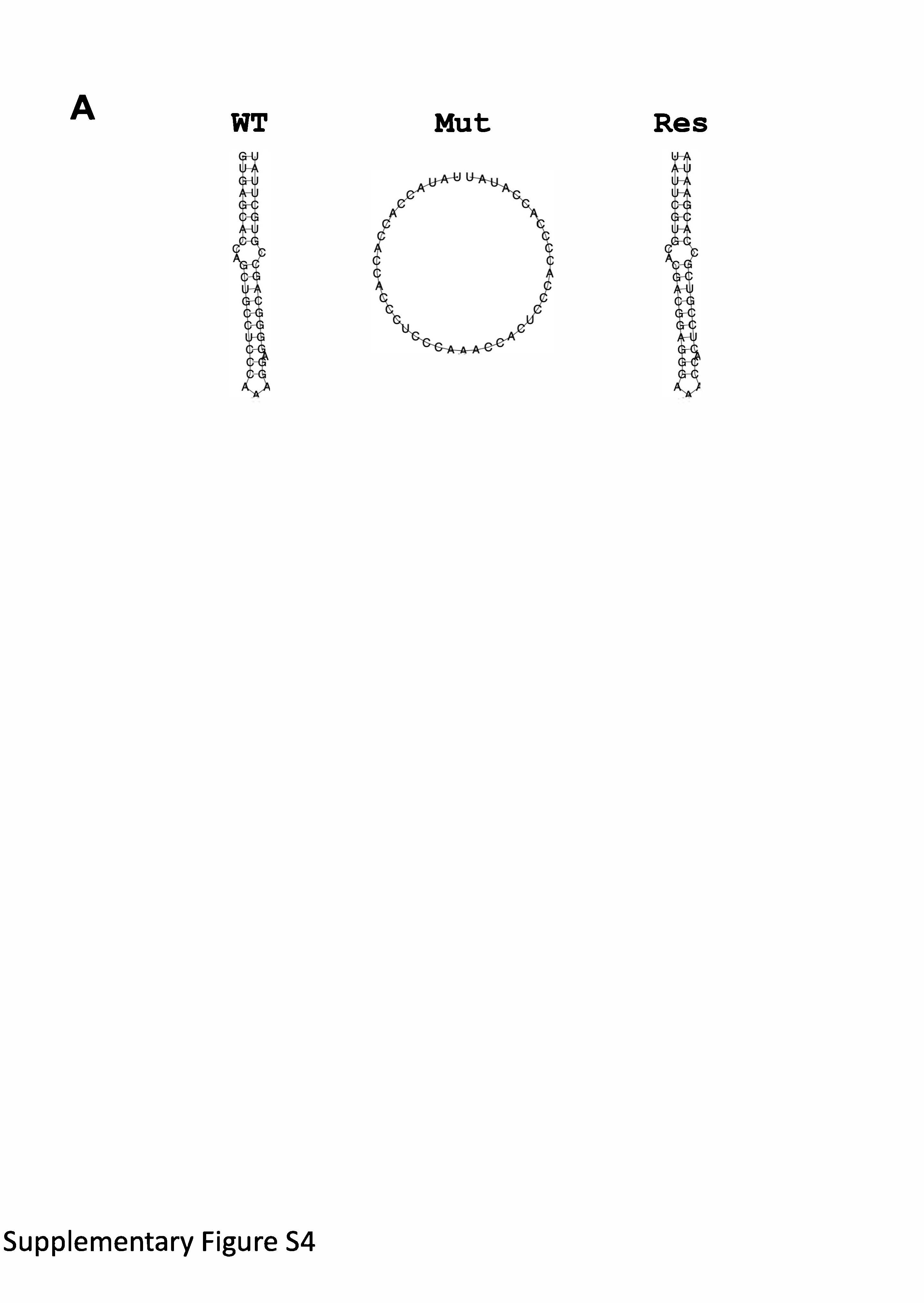

Supplement: Supplementary file 1 [file ijms-23-00215-s001.zip › SupFig4.jpg]

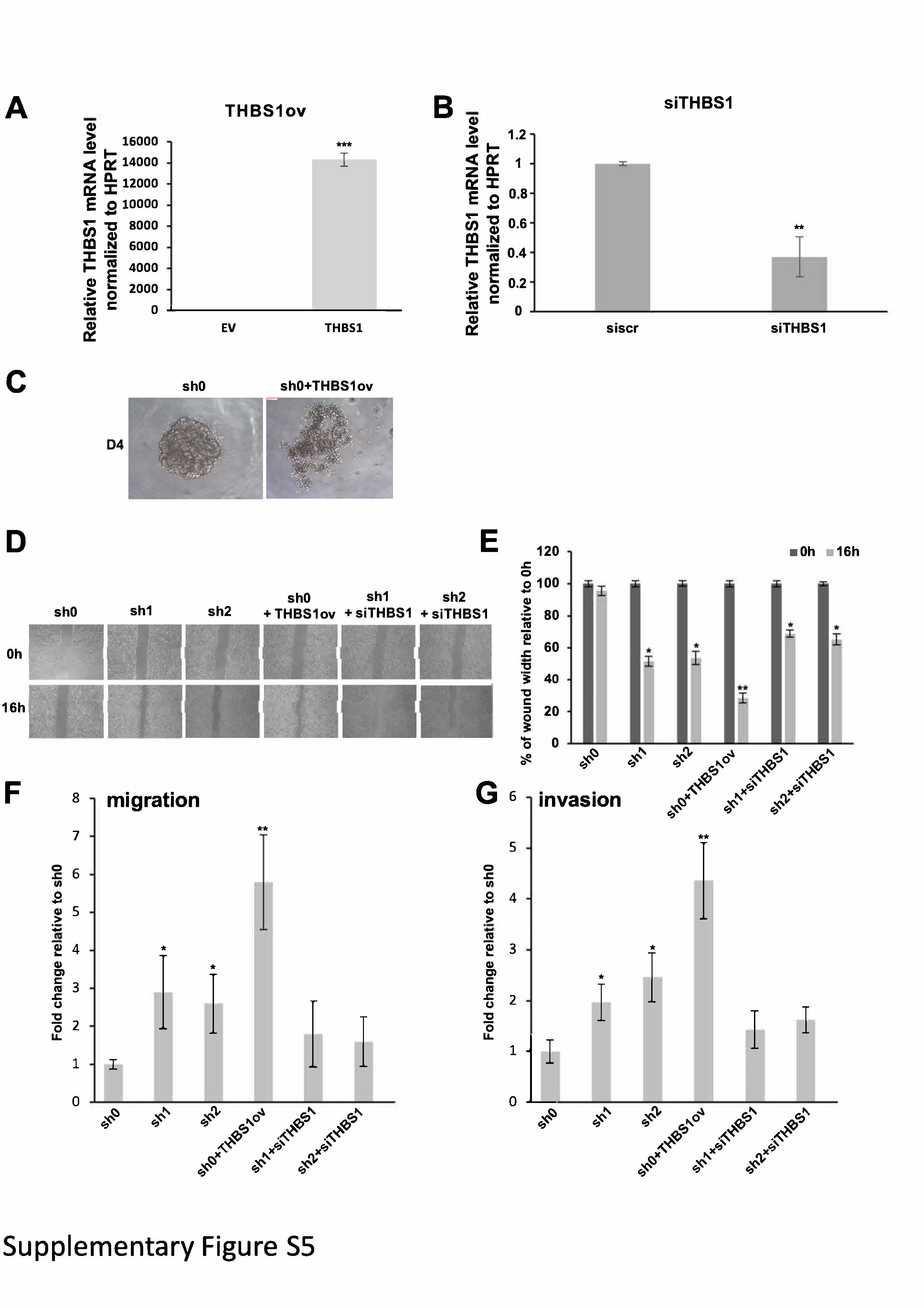

Supplement: Supplementary file 1 [file ijms-23-00215-s001.zip › SupFig5.jpg]
